# Thickness Engineering in HfO_2_-Based Ferroelectrics: A Critical Review of Phase, Switching, Reliability, and Device Trade-Offs

**DOI:** 10.3390/nano16191208

**Published:** 2026-09-24

**Authors:** Yuwei Cai, Yao Li, Zhongjian Gao, Qingzhu Zhang, Zhaohao Zhang, Wenxiu Dong, Wen Ren, Ruige Zhang

**Affiliations:** 1 College of Mechanical and Electrical Engineering, Sanming University, Sanming 365004, China; 2 Key Laboratory of Universities in Fujian Province for Intelligent Control of Equipment, Sanming 365004, China; 3 School of Integrated Circuits, University of Chinese Academy of Sciences, Beijing 100029, China

**Keywords:** Hf_0.5_Zr_0.5_O_2_, thickness scaling, phase stability, ferroelectric switching, reliability, ferroelectric devices

## Abstract

HfO_2_-based ferroelectrics have extended a familiar CMOS high-κ dielectric into a materials platform for nonvolatile memory, low-power logic, and in-memory hardware. This review examines thickness engineering in hafnia, especially Hf_0.5_Zr_0.5_O_2_ (HZO), through the state produced during fabrication, the voltage and time available for switching, and the signal retained during operation. Representative quantitative comparisons retain stack, anneal, electrical state, and endpoint definitions; they show that conditioning and drive voltage can change the apparent thickness trend substantially. Capacitor memory, FeFETs, tunnel junctions, negative-capacitance transistors, and computing devices are compared through architecture-specific read margins and failure criteria. The main unresolved challenge is to isolate thickness effects from coupled process and interface changes and establish reproducible, device-specific operating windows.

## 1. Introduction

HfO_2_ entered advanced CMOS manufacturing as a high-κ dielectric, well before its switchable polarization was recognized. This technological starting point distinguishes HfO_2_ from most established perovskite ferroelectrics when ferroelectricity was first reported in doped HfO_2_ in 2011 [1,2,3]. Subsequent demonstrations in binary ZrO_2_, HfO_2,_ and Hf_1−x_Zr_x_O_2_ solid solutions established that robust polarization behavior could be obtained in films only a few nm thick [4,5]. These developments created opportunities for nonvolatile memory and transistor applications within process environments already familiar with high-permittivity hafnia. Compatibility of the material family, however, does not establish that every ferroelectric stack or thermal process is compatible with a particular CMOS integration flow. The thickness problem arises from the metastability of the functional material. Bulk HfO_2_ is monoclinic and centrosymmetric at ambient conditions, whereas the electrical response of many thin films is associated primarily with a metastable polar orthorhombic phase, commonly assigned to the Pca2_1_ space group. The energy separation among monoclinic, tetragonal, orthorhombic, antipolar, and rhombohedral-like structures is sufficiently small that dopants, grain size, surface energy, electrode constraint, and defects can change the phase assemblage [6,7,8,9,10,11,12]. Reducing thickness changes the balance among these contributions while also changing voltage partition and transport. Consequently, two HZO films of identical physical thickness may differ markedly in phase fraction, local field, vacancy distribution, and switching statistics.

The practical question is therefore which thickness range supplies a reproducible electrical signal under the conditions of the intended device. The useful range is therefore an operating window, not a universal thickness. That window depends on the device. A capacitor memory needs switched charge within its access time; a ferroelectric field-effect transistor (FeFET) needs separated threshold states after writing and retention; a ferroelectric tunnel junction (FTJ) needs a stable polarization-dependent current; and a negative-capacitance field-effect transistor (NC-FET) needs useful gate amplification with acceptable hysteresis and drift [13,14,15,16,17,18,19,20,21]. A large remanent polarization measured slowly cannot by itself establish any of these functions. Pulse-resolved characterization, leakage controls, and state-dependent reliability are needed to connect the material response to device operation [22,23,24,25,26,27]. Representative milestones are shown in Figure 1.

Existing reviews provide detailed accounts of hafnia fundamentals, deposition routes, and device applications [32,35,36,37,38,39]. Here, we organize that knowledge around the consequences of changing physical thickness. The contribution is a critical comparison framework that separates phase selection, electrical thickness, switching-time distributions, and architecture-specific failure. The literature synthesis emphasizes peer-reviewed studies with identifiable thickness and sufficient stack, process, or measurement information to interpret a scaling claim.

The argument proceeds from the state created during fabrication to the signal maintained during operation. Section 2 explains how processing changes the accessible thickness window. Section 3 examines thickness as a structural, electrostatic, and statistical variable. Section 4 and Section 5 address the measurement of switching kinetics and the subsequent evolution of wake-up, fatigue, retention, leakage, and failure. Section 6 then evaluates the cost of thinning or thickening for each architecture.

## 2. Material Processing and Polarization Phase Stabilization

Thickness enters processing through the relative contributions of the film interior and its boundaries. For a fixed lateral area, the bulk free-energy contribution scales with film thickness, while the two interface contributions do not. Grain boundaries, elastic constraint, electrostatics, and kinetic barriers complicate this balance [7,8,9,40,41]. This explains why thickness cannot be optimized after deposition chemistry and electrodes have already been chosen: those choices determine the phase competition to which thickness contributes. Thickness data can only be interpreted in the context of this complete history. The process–structure–property relationships used throughout this review are summarized in Figure 2.

### 2.1. Deposition Routes and the Lower Thickness Boundary

Deposition sets the chemical disorder, density, vacancy population, stress, and nucleation landscape from which the film later crystallizes. Atomic layer deposition (ALD), reactive sputtering, Pulsed laser deposition (PLD), and Chemical solution deposition (CSD) reach this starting point through different chemical and energetic paths, so nominally similar HfO_2_ or HZO films need not develop the same phase assemblage or electrical response [37,42,43,44,45,46,47].

ALD controls the local cation sequence through self-limiting half-cycles. Kim et al. showed that the precursor–oxidant pair affects impurity removal, leakage, and ferroelectric response in HZO [42]. Weeks et al. varied both the HfO_2_/ZrO_2_ nanolaminate period and growth temperature and found that the cycle-defined sequence continued to influence the annealed structure and electrical response [43]. Thus, the nominal number of ALD cycles is insufficient to define the active ferroelectric thickness; measured continuity and post-anneal composition are also needed. Reactive sputtering couples oxygen chemistry to energetic bombardment. In approximately 11 nm HZO, Lee et al. showed that the oxygen fraction changes grain statistics and the phase balance after annealing [44]. When thickness is varied by deposition time, initial bombardment, interfacial oxidation, and stress relaxation may change differently from the film volume. PLD can crystallize the film during growth rather than through a separate amorphous-to-crystalline step. Shimizu et al. deposited 15 nm, 7 mol.% Y-doped HfO_2_ at 700 °C and 10 mTorr O_2_ on an epitaxial ITO/(110)YSZ template [45]. Epitaxial studies provide access to strain and orientation effects that are difficult to isolate in polycrystalline stacks [48,49,50]. Their thickness limits nevertheless depend on the template, growth temperature, oxygen pressure, and cooling history, so they do not transfer directly to an ALD/TiN process. CSD creates the oxide network through solvent removal, ligand decomposition, densification, and crystallization. Starschich et al. used repeated coat–anneal steps to produce Y-doped HfO_2_ films with similar P–E responses from 18 to 70 nm [46]. Mandal et al. subsequently showed that the annealing atmosphere changes the ferroelectric response of solution-processed hafnia [47]. At the thin end, continuity and residual porosity become critical; at the thick end, repeated heat treatments change the crystallization history of previously deposited material. Table 1 relates these route-specific variables to the thickness question rather than ranking the routes by polarization alone.

### 2.2. Thermal Crystallization and the Upper Thickness Boundary

Annealing selects among competing structures while changing grain size, texture, residual stress, and interfacial reaction. A temperature that crystallizes a thicker film may leave a thinner one insufficiently ordered. Conversely, the larger grains and relaxed constraint of a thicker film can favor monoclinic growth. Park et al. examined thickness and annealing temperature together, demonstrating why a processing window should be represented in more than one dimension [51]. Thermal hysteresis in epitaxial films further shows that heating and cooling do not necessarily follow the same structural path [52]. Temperature-dependent phase competition can also alter pyroelectric and energy-storage responses [53].

Interlayers and electrodes participate in this selection. Lederer et al. showed that an Al_2_O_3_ interlayer changes both grain-scale structure and electrical response, demonstrating that the interfaces present during annealing participate in crystallization [54]. Kim et al. obtained large polarization at a reduced thermal budget through TiN-induced stress [55], while Mimura et al. observed thermal hysteresis and an orientation-dependent transition path in epitaxial Y-doped HfO_2_ [52]. Stress-dependent studies reach the same conclusion [56,57,58]: the relevant variable is the complete time–temperature–stress history, not the peak temperature alone. Figure 3 juxtaposes the crystallization sequence and an experimentally resolved thermal transition.

The useful upper thickness boundary is reached when the cost of monoclinic coarsening, stress relaxation, or increased write voltage outweighs any gain in insulation. The lower boundary can be reached through incomplete crystallization before transport becomes dominant. Neither boundary can be assigned from peak annealing temperature alone. Ramp rate, dwell, cooling, ambient, electrode deposition order, and any post-metallization treatment determine how a thickness series should be interpreted.

### 2.3. Composition, Dopants and Laminate Architectures

Ferroelectricity has been reported in Si-, Al-, Y-, La-, Gd- and other doped HfO_2_ systems, as well as across Hf_1−x_Zr_x_O_2_ solid solutions. Dopants and Hf/Zr mixing change phase energies, defects, and grain development [1,2,3,4,5,51]. Their effects depend on thickness because the relative contributions of volume, surfaces, and internal interfaces also change. Calculations show that carrier compensation, stacking direction, and local cation order can modify the polar phase and switching pathway [59,60,61,62,63,64]. These predictions identify possible mechanisms; they do not independently specify the phase fractions obtained after a real deposition and anneal.

Designed laminates introduce a further distinction between total film thickness and individual sublayer thickness. A random HZO alloy and an HfO_2_/ZrO_2_ sequence with the same average composition do not share the same interface density. Cheema et al. demonstrated mixed ferroic order in a gate stack near 2 nm, while more recent structural work has examined phase transitions in hafnia superlattices [31,65]. Composition studies should therefore report the measured cation ratio, dopant distribution, sublayer thickness, interface density, oxygen activity, and thermal history. A useful laminate design must preserve its intended interfaces over the thermal budget of the completed device.

### 2.4. Electrodes and Interfaces: Stress, Screening and Oxygen Exchange

The electrodes are active parts of the ferroelectric stack. They constrain crystallization, screen polarization, exchange oxygen, and set carrier-injection barriers.

Hamouda et al. resolved temperature-dependent chemistry and band alignment at TiN/HZO, confirming that the electrode exchanges oxygen with the film [66]. Replacing the electrode or introducing an oxide interlayer can change the stabilized phase, resistance distribution, or cycling response [67,68,69,70,71]. Figure 4 illustrates how an epitaxial interface termination changes growth and electrical response, together with the associated charge-transfer interpretation.

As thickness decreases, a fixed reaction layer or screening length becomes a larger part of the voltage-bearing stack. A nominally symmetric MFM capacitor can remain chemically asymmetric because the bottom interface experiences deposition and annealing before the top interface is formed. Oxygen-active interfaces can improve cycling stability, as demonstrated by CeO_2−x_-containing HZO heterostructures [26], but the relevant benefit depends on the complete electrode sequence. Interface engineering can move a thickness boundary; it cannot be represented by a correction to nominal thickness alone. These observations explain why a thickness trend can change sign when the electrode material or deposition order changes. The two interfaces are rarely equivalent, and annealing can enlarge their chemical and mechanical asymmetry. A stack description should therefore include electrode material and thickness, deposition order, reaction or interfacial layers, and annealing sequence.

### 2.5. Defect Dynamics, Phase Verification and Reproducibility

Oxygen vacancies and electronic traps link process history to time-dependent electrical behavior. A controlled vacancy population may assist charge compensation and non-monoclinic crystallization, but mobile vacancies can also produce internal bias, trap-assisted conduction, and pinning [12,72,73,74]. Operando observations of oxygen migration and bias-driven structural change provide direct evidence for this coupling [75]. Such results establish a mechanism in the measured stack; however, similar loop shapes in another stack do not establish that the same mechanism is active. Conventional grazing-incidence X-ray diffraction (XRD) provides an ensemble phase constraint, but overlapping orthorhombic and tetragonal reflections, texture, and small diffracting volume limit unique assignments. Transmission electron microscopy (TEM), electron diffraction, four-dimensional scanning TEM (4D-STEM), and electron ptychography can resolve local structure, including oxygen displacements in favorable cases [54,76,77].

Second-harmonic generation (SHG) probes non-centrosymmetric response with symmetry and interface contributions that must be modeled. Piezoresponse force microscopy (PFM) probes local electromechanical behavior, but electrostatic forces, charge injection, and electrochemical strain can complicate interpretation. Neither method alone uniquely identifies a space group. Leakage-corrected electrical switching establishes a functional charge response without specifying crystallographic symmetry. Operando methods are especially useful when they link structural change to bias history, provided temporal resolution and device geometry are stated [30,32,35,75,78]. A persuasive phase assignment combines these complementary constraints and distinguishes a non-monoclinic fraction, a polar distortion, and reversible polarization.

## 3. Thickness Engineering and Ultrathin Scaling

In HfO_2_-based ferroelectrics, thickness changes phase stability, operating voltage, leakage, retention, and variability at the same time. Physical ferroelectric thickness, interfacial-layer thickness, and electrical thickness answer different questions. *t*_FE_ is used for measured physical ferroelectric thickness and *t*_IL_ for a separately identified interfacial dielectric. An electrical thickness is model-dependent and should be reported together with the permittivity or equivalent circuit used to obtain it. Equivalent oxide thickness (EOT) is defined from the measured gate stack capacitance per unit area c_stack_ by EOT = ε_0_ε_SiO2_/c_s_ under stated bias and frequency conditions. In a nonlinear ferroic gate stack, the operating branch, sweep history, frequency, and semiconductor correction matter. Accordingly, an EOT of approximately 0.65 nm in a roughly 2 nm ferroic superlattice does not demonstrate a 0.65 nm physical ferroelectric film [31]. Here, “ultrathin” denotes physical ferroelectric thicknesses below about 8 nm; the sub-5 nm interface-sensitive regime and approximately 1 nm tunnel barrier regime are distinguished where relevant. Furthermore, the 8 nm boundary is an organizational convention, not a universal physical transition or critical thickness. Figure 5 illustrates how structural, electrostatic, and reliability constraints jointly define the practical thickness range for a given stack and electrical history.

### 3.1. A Process-Dependent Thickness Window

A film is useful only over the range in which the desired fluorite-derived state remains stable without excessive transport or interface penalties. Greater thickness can increase switching voltage and grain coarsening; smaller thickness increases leakage, interfacial voltage loss, and statistical variation. Between these limits, the polar-phase fraction, voltage, leakage, and switching-time distribution may all be acceptable [25,30,79].

For a small grain, surface energy can approach the bulk free energy separation among monoclinic, tetragonal-like, and polar orthorhombic states. Electrode confinement and interfacial strain may then suppress the equilibrium monoclinic structure over a finite size range [7,8,35,41]. This is not a monotonic thin-film effect: composition, orientation, oxygen stoichiometry, and mechanical boundary conditions still determine the state that is retained. A trend in P_r_ may consequently include changes in phase fraction, polarization projection, grain connectivity, and interfacial volume. Designed HfO_2_/ZrO_2_ sequencing shifts the same boundaries by increasing interface density [31]. Random alloys, nanolaminates, epitaxial films, and polycrystalline capacitors should therefore be compared only when cation distribution, electrodes, interlayers, texture, annealing, and physical thickness are all known.

Park et al. found reduced pristine polarization with decreasing thickness below 8 nm and substantial polarization increases after wake-up; the thinner films still retained less polarization after conditioning [28]. The earlier thickness–annealing study reported its highest Pr of approximately 16 μC cm^−2^ for a 10 nm HZO film annealed at 600 °C [51]. Epitaxial thickness studies show that orientation and strain can produce a different trend [10,48,50,80]. These observations motivate comparing the full structure and electrical history before attributing a difference in polarization to physical thickness.

### 3.2. Voltage Partition and Distributed Switching Kinetics

Scaling can reduce the terminal voltage needed for switching, but that voltage is divided among the ferroelectric layer, interfacial dielectrics, electrodes, semiconductor depletion, and parasitic resistance. For a specified waveform, the voltage balance is:

(1) $$V_{app}=V_{FE}+\sum_{i}V_{IL,i}+V_{sc}+V_{scr}+{\mathrm{IR}}_{s}$$

Here, V_app_ is the applied voltage, V_FE_ is the ferroelectric voltage, V_IL,i_ is the drop across interfacial layer i, V_sc_ is the semiconductor contribution, V_scr_ represents finite screening contributions, and IR_s_ is the series-resistance drop. V_scr_ denotes the combined electrostatic potential drops associated with finite screening in the metal electrode(s). Its value depends on the adopted screening model and cannot be determined from the terminal voltage alone. It is distinguished from the voltage drops across physical interfacial dielectrics, V_IL,i_, and within the semiconductor, V_sc_. If an effective interface capacitance already includes electrode screening, the same contribution must not be added again as a separate V_scr_ term. The voltage decomposition, including the treatment of trapped charge and built-in potential offsets, must be specified consistently. The internal field can be approximated by V_FE_/t_FE_ only when spatial variations within the ferroelectric can be neglected. The field in the active layer may therefore differ substantially from the nominal voltage divided by physical thickness. Sawabe et al. found that thickness changes the distribution of switching times across field and pulse width rather than simply shifting one coercive voltage [25]. This links voltage partition to the slow fraction of the switching population. It also explains why a reduction in terminal switching voltage need not imply a reduction in intrinsic coercivity, and why comparing transients at a common terminal voltage differs from comparing them at a common estimated E_FE_. A lower terminal voltage is useful only if the complete stack still switches the slow tail of the distribution. Kinetic data should give the delivered waveform, active area, initial state, pulse history, integration window, device count, and the distribution relevant to the intended write-error rate. Figure 6 compares reported Pr and cycling endpoints as a function of thickness.

Figure 6a expresses remanent polarization on a common Pr axis by dividing explicitly reported 2Pr values by two, while preserving the distinction between pristine and conditioned states. The Park et al. results illustrate how field cycling changes polarization at a fixed nominal thickness [28]. The additional literature reveals substantial variation among devices with comparable thicknesses: reported Pr values span approximately 9.5–34 μC cm^−2^ at 5 nm and 3–26 μC cm^−2^ at 10 nm. This spread encompasses differences in electrodes, interfaces, substrates, thermal processing, measurement protocols, and electrical history. Converting the reported polarization quantity therefore facilitates comparison without making these measurements equivalent in all other respects.

Figure 6b distinguishes observed breakdown from cycling lower bounds, identified by five-point stars. Comparisons within individual studies show how strongly the reported cycling performance depends on operating conditions and stack design. For the 4.6 nm devices reported by Lyu et al. [81], cycling reached 10^11^ cycles at 2.5 V without observed breakdown, whereas breakdown occurred near 10^10^ and 10^9^ cycles at 3.0 and 3.5 V, respectively. For Lyu et al. [82], the plotted values are the last explicitly documented switching points before subsequently reported breakdown; they are not failure-free censored test endpoints. Furthermore, Kim et al. demonstrated 5 nm HZO capacitors with a reported Pr of approximately 10 μC cm^−2^ at 1.0 V and endurance exceeding 10^10^ cycles under a cycling voltage of 1.2 V. These results show that appreciable polarization and extended cycling endurance can coexist at reduced thickness, although the polarization and endurance values correspond to different applied voltages [83]. Wang et al. extended this comparison to 3 nm HZO, reporting Pr values of approximately 24.8 and 10.8 μC cm^−2^ at thicknesses of 6 and 3 nm, respectively. The 3 nm films exhibited a saturation voltage of approximately 0.7 V and sustained approximately 10^11^ cycles without breakdown; the saturation voltage should not be interpreted as the endurance-test voltage. Their observation of pronounced wake-up below 5 nm further indicates that the usable polarization of ultrathin films remains sensitive to electrical conditioning [84]. Hsain et al. data illustrate the influence of interfaces at a fixed HZO thickness: under a reported field amplitude of 3.5 MV cm^−1^ and a cycling frequency of 100 kHz, the 10 nm control devices broke down near 2 × 10^4^ cycles, while the alumina-interlayer variants reached 10^8^ cycles without observed breakdown [85]. These cycling lower bounds establish the demonstrated test duration without observed breakdown; they do not establish an equally long functional lifetime. Polarization degradation and the remaining readable signal must be assessed against a stated acceptance criterion.

The compilation thus reinforces the importance of evaluating thickness together with the device stack and electrical history. A practical thickness window must satisfy the required polarization or switching signal within specified voltage, retention, and cycling constraints. The broader literature coverage provides more examples of achievable performance, but the scatter does not isolate a universal thickness dependence or establish a common optimum.

**Figure 6:**
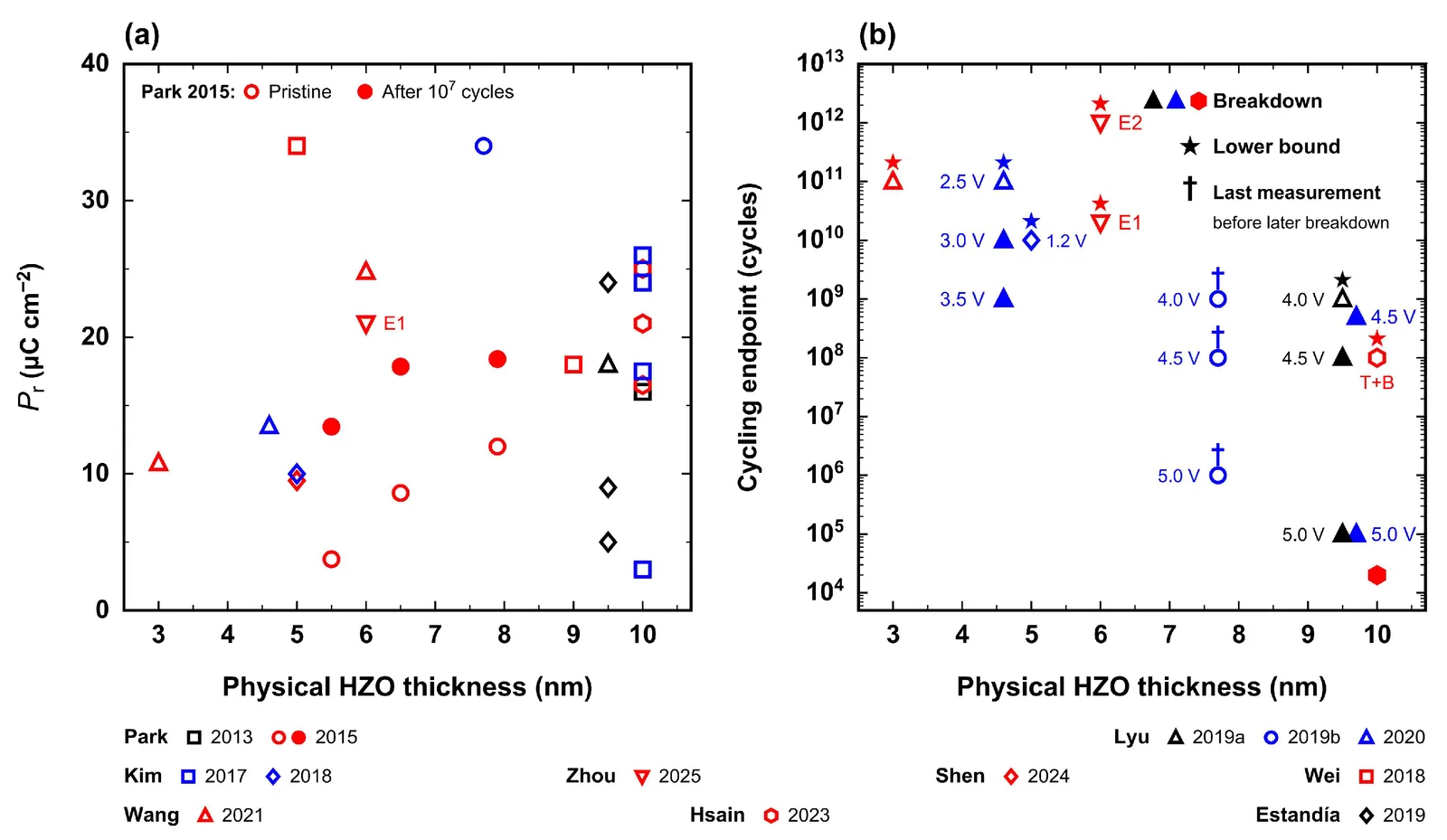
Summary of representative literature data for HZO: (**a**) remanent polarization Pr versus physical thickness and (**b**) cycling endpoint versus physical thickness. Symbols identify individual studies by author and year. Sources: Park [28,51], Lyu [81,82,86], Kim [55,83], Zhou [26], Shen [87], Wei [10], Wang [84], Hsain [85], and Estandia [48]. Lyu 2019a and 2019b correspond to Refs. [86] and [82], respectively. Explicitly reported 2Pr values are divided by two. In (**a**), open and filled red circles for Park et al. [28] denote pristine and 107-cycle-conditioned states, respectively; this convention applies only to that dataset. In (**b**) filled black/blue triangles (Lyu) and the filled red hexagon (Hsain) indicate reported breakdown, whereas five point stars identify reported cycling lower bounds, including test endpoints reached without observed breakdown. Daggers (†) identify the last documented switching measurements for Lyu et al. [82] before subsequently reported breakdown; these are not failure-free censored test endpoints. Cycle counts are read from the associated study symbols; the vertically offset stars and daggers are status annotations. E1 and E2 denote the CeO_2−x_-capped and additional top LSMO interface designs of Zhou et al. [26], respectively. T + B denotes coincident results for separate top- and bottom-alumina-interlayer variants reported by Hsain et al. [85]. This is a literature compilation, not a controlled thickness series: stacks, electrodes, annealing conditions, and electrical histories differ. Cycling lower bounds do not establish degradation-free operation or a common functional lifetime, and the compilation does not isolate a universal thickness dependence.

### 3.3. Leakage, Depolarization and Retention Under Cycling

Direct tunneling, defect-assisted conduction, and local breakdown become more likely as the barrier is thinned; roughness or one defective grain can then dominate the current. At the same time, finite screening and low-permittivity interlayers increase the depolarization field, promoting back-switching or apparent polarization loss [88,89,90]. A lower write voltage is beneficial only if the two written states remain distinguishable from leakage throughout the required cycling and retention history. The associated structural, electrostatic, transport, and endurance trade-offs are summarized in Figure 7.

The data compiled in Ref. [88] make clear that operating voltage alone does not define the ultrathin limit. A thinner film lowers terminal voltage but increases tunneling, tightens the crystallization budget, and changes the margin between coercive and breakdown fields. Retention is then controlled by screening, carrier injection, interfacial permittivity, defect motion, and write history. Both polarization states should be read after defined pulses and delays, and leakage should be monitored during cycling rather than only before and after stress.

### 3.4. Grain Statistics and Architecture-Specific Ultrathin Limits

Lateral area determines how many grains a device samples, while thickness can change grain size, texture, and the fraction of inactive material. For approximately columnar grains, a geometric estimate is:

(2) $$N_{g}\approx \frac{4A}{{{\pi d}_{g}}^{2}}$$

N_g_ is the geometric grain count, A is the active area, and d_g_ is an effective lateral grain diameter. This is a calculated packing estimate, not a measured population of independently switching domains. A geometric grain can contain multiple switching regions, while correlations can couple switching across neighboring grains; neither the number nor the statistical independence of switching regions follows from Ng alone. Francois et al. showed that lateral scaling exposes grain-level distributions in BEOL-compatible HZO and Si:HfO_2_ capacitors [79]. Buragohain et al. directly mapped nonuniform domain activation [91]. Transient measurements show that the distribution of activation fields or switching times can broaden even when low-frequency P_r_ changes little [25,29]. Interface asymmetry, fewer grains, and larger local field variations create a slow tail that sets the pulse width needed for a given write-error rate. The relevant statistical description therefore includes lithographic area and an effective switching population constrained by electrical statistics or local observations, rather than equating that population with Ng. Mean polarization cannot describe the tails that determine write error, leakage, and early failure. Thickness and area series should include distributions of charge, switching time, retention, and endurance together with local structural statistics and device count.

Below about 5 nm, interface dipoles, atomic-scale roughness, finite screening, and tunneling become part of the active device rather than small corrections. Lee et al. associated scale-free ferroelectricity in HfO_2_ with localized polar modes, and Cheema et al. demonstrated functional HfO_2_–ZrO_2_ interfaces near gate-oxide thicknesses [30,31,78]. Shen et al. investigated transferred 1–5 nm HZO membranes as well as epitaxial films [87]. Their 5 nm epitaxial film yielded a leakage-corrected PUND 2Pr near 19 μC cm^−2^, while measurements of thinner epitaxial films were strongly affected by leakage. The membrane experiment changes the interfaces and mechanical boundary conditions. It must not be used as evidence that an otherwise unchanged 1 nm transistor gate stack has identical retention or leakage. Optimization at this scale should begin with the required output—switched charge, threshold shift, tunnel current, or voltage gain and then test leakage, variability, and reliability in the corresponding MFM, MFIS, or tunnel stack. Table 2 compiles several representative studies, and Table 3 contains the corresponding electrical metadata for the same studies.

## 4. Thickness-Dependent Switching Dynamics and Frequency Response

The structural and electrostatic constraints discussed in Section 3 also determine how much polarization can be reversed within a finite pulse. Reducing the physical ferroelectric thickness lowers the voltage required to produce a given field only when voltage partitioning is accounted for; it does not guarantee faster or more complete switching. Interfaces, grain structure, and electrical history can change the distribution of local switching times as the film becomes thinner. Dynamic characterization must therefore connect the waveform delivered to the device with the switched charge available at the circuit timescale. Figure 8 outlines the measurements needed to separate that charge from displacement, leakage, trapping, and test-path artifacts.

### 4.1. Distributed Switching and Model Limits

Polycrystalline HZO contains grains and local regions with different orientation, phase fraction, defect density, interface condition, and internal field. Under an applied pulse, some regions reverse rapidly while others require a larger field or longer time. The measured switching current is the sum of these local events. A large low-frequency P_r_ can consequently coexist with incomplete reversal under the nanosecond-to-microsecond pulses used by memory circuits.

Kolmogorov–Avrami–Ishibashi descriptions represent reversal as nucleation and domain growth with a characteristic time and an effective dimensionality. They are useful when switching regions are statistically similar. Hafnia films often require a distribution of switching times or activation fields, which is captured phenomenologically by the nucleation-limited switching (NLS) framework [29,91,92]. A common field dependence follows a Merz-type relation:

(3) $$\tau \left(E\right)=\tau_{0}exp\left(\frac{E_{a}}{E}\right)$$

where $\tau_{0}$ is a fitted time prefactor and $E_{a}$ is an effective activation field. The macroscopic switched polarization may then be expressed as an integral over a distribution $g\left(log\tau \right)$:

(4) $$\Delta P\left(t,E\right)=2P_{r}\int \left[1-exp\left(-{\left(\frac{t}{\tau }\right)}^{n}\right)\right]g\left(log\tau \right)\;d\left(log\tau \right)$$

where ΔP is the polarization change after a pulse of duration t, τ is a local characteristic switching time, and n is an effective growth exponent. E denotes the estimated ferroelectric field magnitude. In Equation (4), log τ abbreviates u = log_10_(τ/τ_ref_), with τ_ref_ = 1 s. The non-negative distribution g(u; E) is a probability density per unit u (per decade of switching time), normalized over −∞ < u < ∞ so that ∫g(u; E) du = 1. Its dependence on E is suppressed in Equation (4), and the same logarithm convention must be used in the distribution and integration measure. The factor 2Pr applies only to complete reversal between symmetric remanent states. For asymmetric or incompletely accessible switching states, it is replaced by the measured saturated switched-charge density ΔP_sat_ for the relevant polarity, preset state, and reference pulse; for complete reversal between two remanent states, ΔP_sat_ = |P_r_^+^ − P_r_^−^|. The amplitude and time distribution should be specified separately for each switching polarity. In addition, Equation (4) is a phenomenological ensemble representation, not a universal microscopic switching model. The fitted distribution does not directly resolve individual domains, and different parameter sets may reproduce the same data. Independent variation in field, pulse width, and temperature can help constrain the model but does not by itself guarantee parameter uniqueness.

These equations are explanatory phenomenological models; no new microscopic parameters are extracted here. A good fit alone cannot distinguish independent nucleation, domain-wall motion, and an instrumental response with a similar time dependence [76,93,94,95]. Local structure and independently measured waveforms are needed to constrain a microscopic interpretation. Figure 9 shows the thickness-dependent switching distributions discussed by Sawabe et al. and Kobayashi et al. [25,58]. The thinner films display broader activation-field distributions and more pronounced program/erase asymmetry. Lee et al. measured reproducible intermediate polarization states together with their statistical spread [92]. Max et al. showed that polarization reversal and vacancy-mediated resistive switching can coexist in doped crystalline HfO_2_ [96]. A multilevel response should therefore be followed through relaxation, read disturb, and cycling before the intermediate current or threshold is assigned entirely to a switched domain fraction.

### 4.2. Frequency Response and the Limits of PUND Subtraction

A frequency trend can be attributed to material kinetics only after the bandwidth and impedance of the test path are known. Lancaster et al. used a modified PUND sequence with a controlled zero-bias delay to quantify polarization loss after switching in HZO and HZO/Al_2_O_3_ capacitors [23], as illustrated in Figure 10a. Cai et al. investigated FinFET time-domain characteristics using pulse train measurements [97]; their separate CMOS-inverter study evaluated nanosecond transient operation [98], using the measurement scheme shown in Figure 10b. Reports should give the waveform at the device, rise and fall times, capacitance, and thermal state so that an instrument limit is not mistaken for a material limit.

Frequency is not a direct proxy for switching speed. A triangular loop at 1 MHz, a 1 μs rectangular PUND pulse, and a small-signal capacitance measurement at 1 MHz probe different processes. Sweep-rate dependence concerns periodic large-signal response; pulse-width dependence probes irreversible reversal; and small-signal capacitance measures the local reversible response about a bias point. Grimley et al. resolved orthorhombic domain walls and orthorhombic–monoclinic interphase boundaries [76]. Alessandri et al. extracted partial reversal with a conditioning-pulse-read sequence [93]. Calculated migration paths also differ among domain-wall structures and topological pathways [94,95]. A microscopic claim is strongest when transient fitting, local structure, and atomistic energetics agree. In practice, the voltage at the device, integration window, and equivalent circuit must be measured, and displacement, leakage, and trapping currents must be separated across area, polarity, temperature, and electrical state.

### 4.3. Comparing Switching Times Across Thicknesses

A thickness-dependent switching-time comparison must separate the voltage benefit of thinning from changes in the switching population. At a common terminal voltage, a thinner film may receive a larger driving field, but interfacial voltage loss can offset that gain. Comparison at a common estimated ferroelectric field instead tests how microstructure and interfaces affect reversal, subject to the voltage partition uncertainty described in Section 3.2. Both comparisons are useful: the first evaluates a circuit voltage budget, while the second helps interpret the distributed kinetics discussed in Section 4.1 [25,29,93]. Neither isolates thickness if electrodes, annealing, composition, or conditioning also change.

For each thickness, switched-charge maps versus pulse amplitude and duration should establish the onset, central response, and approach to completion. Define t50 and t90 as the durations required to reach 50% and 90% of a saturated reference switched charge under an explicitly stated reference pulse and electrical state. Reporting both times reveals a slow tail that the current-peak position can miss. The absolute charge or charge density must accompany these fractions: reaching 90% of a small switchable population may still provide insufficient readout. Positive and negative switching should use the same definition and equivalent presetting, with leakage and subtraction uncertainties retained [23,24]. The shortest measured time is meaningful only if the delivered pulse is resolved. At fixed area, thinning can increase stack capacitance and hence the approximate charging time R_s_C_stack_, even when the intrinsic switching becomes faster. Reconstruct the ferroelectric voltage using the stack model rather than dividing terminal voltage by film thickness. Pulse spacing, integration window, read delay, temperature, active area, device count, and cycling history are also needed to distinguish material kinetics from circuit charging and state evolution.

Finally, repeat the voltage duration maps after conditioning and electrical stress, preserving the initial reference charge alongside any normalization to the stressed state. Otherwise, fatigue can appear as faster switching simply because less charge remains available. For the memories discussed in Section 6, the practical write time is the time needed to obtain a readable charge, threshold, or resistance state at a specified read delay and write-error probability; capacitor t90 alone does not establish it.

## 5. Thickness-Dependent Reliability and Functional Qualification

Thickness scaling changes the reliability problem through local field, leakage, screening, and the volume influenced by interfaces. At the same terminal voltage, a thinner film may experience greater electrical stress; at the same estimated ferroelectric field, different interfacial voltage drops and defect populations can still produce different degradation rates. Wake-up increases the accessible switched charge, fatigue reduces it, and recovery restores part of a degraded response under a stated treatment. Imprint shifts the preferred switching direction, whereas retention loss reduces the stability of a written state during storage. Hard breakdown is an irreversible loss of insulation and is distinct from all of these functional changes. Figure 11 organizes this evolution without implying that every device follows the same sequence.

### 5.1. Wake-Up: Field-Driven Activation of the Switching Population

Wake-up is most usefully described as an increase in the switching population accessible to the measurement. It need not arise from one microscopic mechanism. Proposed mechanisms include redistribution of charged defects, changes in internal bias, depinning, and phase evolution [22,75,99]. A pinched loop becoming more open does not identify which mechanism dominates. Epitaxial ZrO_2_ without conventional wake-up further shows that conditioning requirements depend on the initial structure and interfaces [49].

Thinner films can make conditioning more consequential because a small interfacial or inactive fraction represents a larger part of the active volume. However, the direction of the effect is process-dependent. The Park et al. data in Figure 6a show why pristine and conditioned polarization must remain separate [28]. The conditioning cost includes pulse amplitude, duration, duty cycle, temperature, and total exposure, not just cycle count. A useful device must either meet its signal requirement initially or reach it through a conditioning procedure consistent with its voltage and lifetime budget. A phase- or defect-based explanation of wake-up also requires local or structural evidence.

### 5.2. Fatigue and Interface-Controlled Endurance

Fatigue denotes progressive loss of functional signal under repeated operation, while recovery denotes restoration under a defined additional treatment. Oxygen-active capping, bottom-electrode modification, and interfacial evolution can change both processes [100,101,102,103]. Figure 12 presents cycling, leakage, and rejuvenation together with the source study’s structural interpretation [104]. Recovery is evidence that some degradation is reversible; it does not imply that all damage has been removed or that the recovery operation is free of energy and lifetime costs. For thickness comparisons, the stress must be stated as voltage, estimated field, pulse width, polarity, duty cycle, and temperature. The 4.6 nm voltage-dependent endpoints in Figure 6b demonstrate how strongly the result can change without any change in thickness [81]. Interface-engineered 6 nm HZO provides a separate example of a high cycling lower bound [26]. Such endpoints should remain attached to their signal thresholds and measurement conditions; they cannot be ranked solely by the largest reported cycle count.

### 5.3. Retention and Imprint: Stability of Both Written States

Retention measures whether the required distinction between written states persists with time. Imprint describes a preference for one polarization direction or a shift in the switching characteristic. Depolarization, trapping, and defect redistribution can affect both [89,90]. A smaller depolarization contribution does not guarantee better two-state retention if charge trapping increases the internal asymmetry.

Ref. [23] shows delay-dependent polarization loss in an explicitly defined pulse experiment. This emphasizes that read delay is part of the measured state. Retention should be evaluated after specified program and erase pulses, at stated temperatures and cycling states, with read bias and read disturbance controlled. Direct observations must be separated from extrapolations, and both branches or state distributions should be reported. A fit over short times is insufficient to establish the absence of an additional long-time mechanism. Scaling makes the relation between retained polarization and readout architecture especially important. A capacitor can retain switchable charge while a FeFET loses its threshold window through traps or interfacial leakage [105,106]. A tunnel device can retain polarization while its read-current distribution broadens. The signal available to the sensing circuit therefore determines whether a measured drift is tolerable.

### 5.4. Statistical Failure and Reliability Qualification

Leakage, current dispersion, and local structural changes often appear before the polarization loop collapses. Mulaosmanovic et al. treated retention, imprint, and fatigue as separate reliability modes with architecture-dependent criteria [27]. Shanker et al. examined EOT scaling and bias-temperature instability in a negative-capacitance superlattice [107], whereas Yurchuk et al. showed that trapping and gate-stack leakage can degrade a one-transistor memory even while its ferroelectric capacitor remains switchable [98]. Random-telegraph noise and threshold-voltage variation further connect nanodomain behavior to device distributions [108,109]. Functional failure may therefore precede the loss of a polarization loop. Reports should include leakage during cycling, compliance, area, device count, censoring, and the electrical failure criterion; a median cycle count alone hides early failures and distribution tails.

Furthermore, reliability tests should recreate the electrical history that produces the state being qualified. The workflow in Figure 13 keeps the initial state, stress waveform, remaining memory signal, parasitic responses, device statistics, and application-specific failure criterion in the same comparison.

## 6. Thickness Trade-Offs in Device Architectures

The previous sections establish the material state, operating field, accessible switching fraction, and failure criterion. Device selection brings these constraints together. A single HZO stack is not optimal for every circuit. FeRAM is limited by charge delivered within a sensing interval; FeFET memory by the separation and retention of two threshold states; FTJs by tunneling electroresistance and read current; and NC-FETs by voltage amplification with limited hysteresis. Array and neuromorphic systems add constraints on uniformity and analog update behavior. Materials results must therefore be judged against the metric of the intended architecture [14,38,39]. Table 4 compares the benefits and costs of changing physical ferroelectric thickness. The trends are conditional on the stack: sub-8 nm capacitors, sub-5 nm gate stacks, and approximately 1 nm barriers must be assessed in their own structural and electrostatic regimes, rather than treated as one continuous device-scaling series. Switchable polarization or a hysteretic transfer curve alone does not establish two retained FeFET memory states. Transient voltage gain is a logic-response metric and, by itself, does not establish a nonvolatile memory function.

### 6.1. Ferroelectric Capacitors and FeRAM

In an MFM capacitor, the switched signal is approximately Qsw = A ΔPsw at the specified pulse width. There is no additional multiplicative thickness factor. Thickening increases useful charge only if it improves the switchable fraction or its retention under the operating pulse. It can also suppress direct tunneling, but usually increases the terminal voltage required for a comparable field. Thinning can reduce that voltage while increasing interfacial voltage loss, leakage, or the slow fraction of the switching population [25,79].

Integrated 1T1C FeRAM demonstrations establish the importance of access-device and sensing constraints [13,18]. At a fixed bit area, the thickness choice must satisfy the delivered-charge requirement while respecting access time, leakage during read/write, retention, and endurance. A large low-frequency loop is useful only when it survives these constraints. Conformal deposition in a three-dimensional capacitor can increase area without increasing footprint, but it adds thickness-uniformity and sidewall-crystallization requirements.

### 6.2. FeFET Memory

FeFETs convert polarization and electrostatic boundary conditions into a threshold shift. Reducing tFE can improve gate coupling and reduce physical stack height, but a fixed interfacial dielectric then takes a larger share of the applied voltage. Depolarization, trapping, and local thickness variations can become more important. A thicker ferroelectric can support a larger field-dependent threshold window, yet increases gate-stack electrical thickness and program-voltage cost. The memory window is not proportional to ferroelectric thickness or polarization in a stack-independent way.

Hafnia FeFETs have been demonstrated from early CMOS-compatible structures to 20 nm Si:HfO_2_ devices and 28 nm high-κ metal-gate integration [2,110,111]. Other work reported 100 ns programming and a 2.44 V window in a superlattice Fe-FinFET [112]. These examples establish different operating points; they do not form a controlled thickness series. Device and write-pulse studies show that the accessible window also depends on pulse history and readout protocol [113,114,115]. Memory-window measurements contain polarization and charge-trapping contributions, potentially with different time constants and signs [105,106,116]. Both threshold distributions should therefore be measured after specified program/erase pulses and read delays, including retention and disturb. Figure 14 illustrates conductance dynamic-range statistics versus channel length in an oxide-channel synaptic transistor [117]. These data demonstrate lateral-size-dependent device variability; they do not isolate a ferroelectric-thickness effect. Its relevance to thickness engineering is that channel geometry must be held fixed, or its influence explicitly accounted for, when comparing devices with different ferroelectric thicknesses. The update and read protocols must also be specified, and any differences considered before changes in conductance dynamic range can be attributed to ferroelectric thickness alone.

### 6.3. FTJ and NC-FET: Distinct Outputs from Ultrathin Electrostatics

An FTJ requires a barrier thin enough for measurable tunneling while preserving a reversible polar state. In a simple direct-tunneling model, current depends exponentially on thickness through a barrier decay constant. This makes read current particularly sensitive to roughness and local thickness variation. Thickening can suppress current below the sensing limit; excessive thinning can increase leakage, read disturb, or competing defect-mediated transport [15,20,118]. A resistance change alone is insufficient to distinguish polarization-controlled tunneling from trapping or filamentary switching.

NC-FETs impose a different objective: the nonlinear ferroelectric response must be compatible with the positive capacitance of the interfacial dielectric and channel [19,21]. In an ideal single-domain quasistatic description, stability requires a positive total differential inverse capacitance. Thickness changes the ferroelectric contribution to that series condition, so reducing EOT does not by itself establish stable, hysteresis-free amplification. Domain formation, rate dependence, trapping, and circuit loading can invalidate a simple capacitance-matching estimate.

Studies of 3 nm-HZO FinFETs, back-gate-coupled devices, and related CMOS circuits show useful transistor responses but also dependence on sweep range, pulse sequence, cycling, and bias-temperature stress [97,98,107,108,119,120,121,122,123,124,125]. The comparison rule is explicit: improved NC-FET transfer characteristics or transient voltage gain do not establish two retained FeFET memory states. NC-FET results require suitable non-ferroelectric controls, waveform-dependent measurements, and a stated stability criterion; FeFET memory additionally requires retained state separation.

### 6.4. FeNAND, Logic-in-Memory, and Associative Search

In a vertical FeNAND string, ferroelectric thickness affects both individual cell programming and electrostatic coupling along the string. Thinning can reduce radial stack thickness and programming voltage, but may narrow the margin between intended switching and pass/read disturbance. Thickening can increase the required pass and program voltages and complicate uniform switching along a conformal sidewall. Interlayer engineering and low-power NAND demonstrations address this joint cell-and-string problem [16,17,33,34]. They should be compared through the distributions of cell thresholds, program inhibit, read disturb, and multilevel retention along a string.

Logic-in-memory and associative-search circuits reuse stored states to separate output-current distributions. Their thickness requirement therefore follows from state separation after drift and disturb, rather than from a generic preference for low voltage. Reconfigurable logic, ambipolar transistors, and ferroelectric-diode content-addressable memory provide architecture examples [118,126,127,128,129,130,131]. The decisive comparison is whether thinner devices preserve match/mismatch separation, energy, and yield at the intended array size. Claims of a thickness optimum require a controlled thickness sweep; an isolated circuit demonstration establishes feasibility only.

### 6.5. Analog and Neuromorphic Computing

Analog operation deliberately uses partial state changes. Thinning may lower pulse voltage and energy, while increasing interfacial sensitivity or broadening switching-time distributions. It does not necessarily reduce the number of lateral switching grains at fixed area, as discussed in Section 3.4. Controlled gradual updates may benefit from many independently addressable regions; correlations and a small effective population instead produce large, irregular steps [92,132,133,134,135,136,137]. The appropriate thickness window is defined by update linearity, symmetry, dynamic range, retention, endurance, and device-to-device variation.

Mixed mechanisms can be useful if identified correctly. Halter et al. used fast ferroelectric and slower ionic processes in an oxide-channel synaptic device [117]. Such an analog response should be described as a combination of mechanisms rather than as proof that all intermediate states are partially switched polarization. Broader device-to-system studies show why nonlinearity, state drift, and variability must enter the computing assessment [134,137,138]. The most useful experiment combines a controlled thickness series with the same update protocol and evaluates its effect on a fixed sensing or learning task.

## 7. Conclusions and Outlook

Thickness engineering in hafnia is the selection of a usable operating window under coupled structural, electrostatic, dynamic, and reliability constraints. The quantitative examples show that conditioning can substantially shift polarization at fixed thickness, that interlayers can shift reliability at fixed HZO thickness, and that cycling voltage can move an observed breakdown endpoint by orders of magnitude. These are reasons to retain stack and protocol metadata, rather than to interpret all reported values as a single thickness law.

The first unresolved question is how much of an observed scaling trend remains when annealing, electrodes, electrical conditioning, and measurement field are independently controlled. Factorial thickness–anneal and thickness–interface studies are needed alongside simple thickness series. They should measure both structural state and electrical response so that thickness-induced grain evolution can be distinguished from a changed fabrication recipe.

The second question concerns voltage and active volume near the interface-dominated limit. Physical thickness, capacitance-derived EOT, local voltage drop, and switchable phase fraction must be measured or modeled separately.

The third concerns statistics: the relation among geometric grain count, correlated switching units, and the rare failures that determine array yield remains insufficiently constrained. Larger, explicitly reported device populations and measurements across dies and wafers are necessary to connect nanoscale mechanisms to manufacturing variation.

The fourth question is whether a favorable initial state survives realistic short-write, read-disturb, retention, and recovery histories at the allowed temperature and voltage. Priority should be given to measuring these properties on the same stack and, where feasible, the same devices.

For future thickness comparisons, a minimum reporting checklist is: (i) composition, electrodes, physical ferroelectric and interfacial-layer thicknesses, and deposition/annealing conditions, with EOT reported separately; (ii) the delivered write/read waveforms, voltage-partition model, bandwidth, temperature, leakage, and conditioning history; (iii) active area, device count and distributions of the relevant switching and reliability metrics; and (iv) a functional failure criterion tied to the remaining readable signal, including read delay and retention conditions. Cycling lower bounds, observed functional degradation, and breakdown should be identified separately, and measured retention should be distinguished from extrapolation.

## Figures and Tables

**Figure 1:**
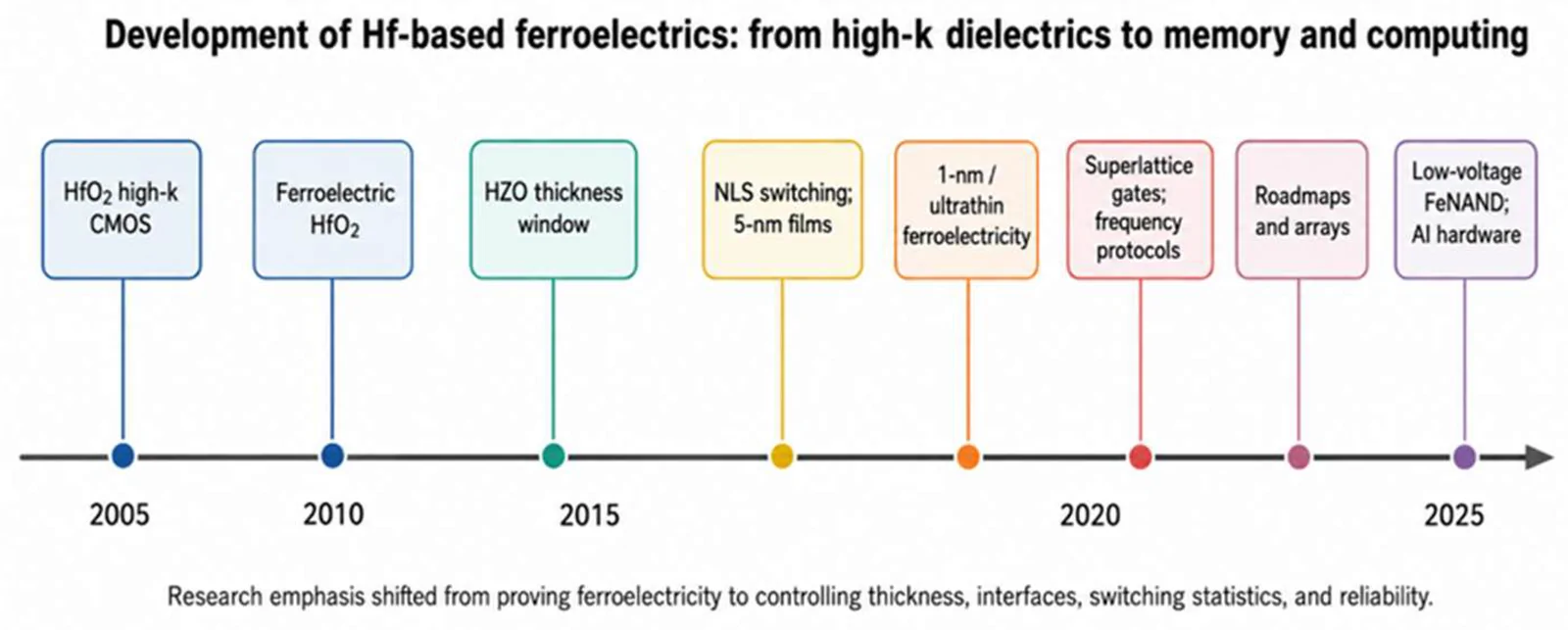
Development of HfO_2_-based ferroelectrics from a CMOS high-κ dielectric to ferroelectric devices. Ferroelectric hafnia was reported in 2011 [1,2,3], sub-8 nm thickness and wake-up behavior in 2015 [28], NLS in 2018 [29], approximately 1 nm ferroelectric films in 2020 [30], and mixed-ferroic superlattice gates in 2022 [31]. Recent roadmaps and FeNAND demonstrations are represented by Refs. [32,33,34].

**Figure 2:**
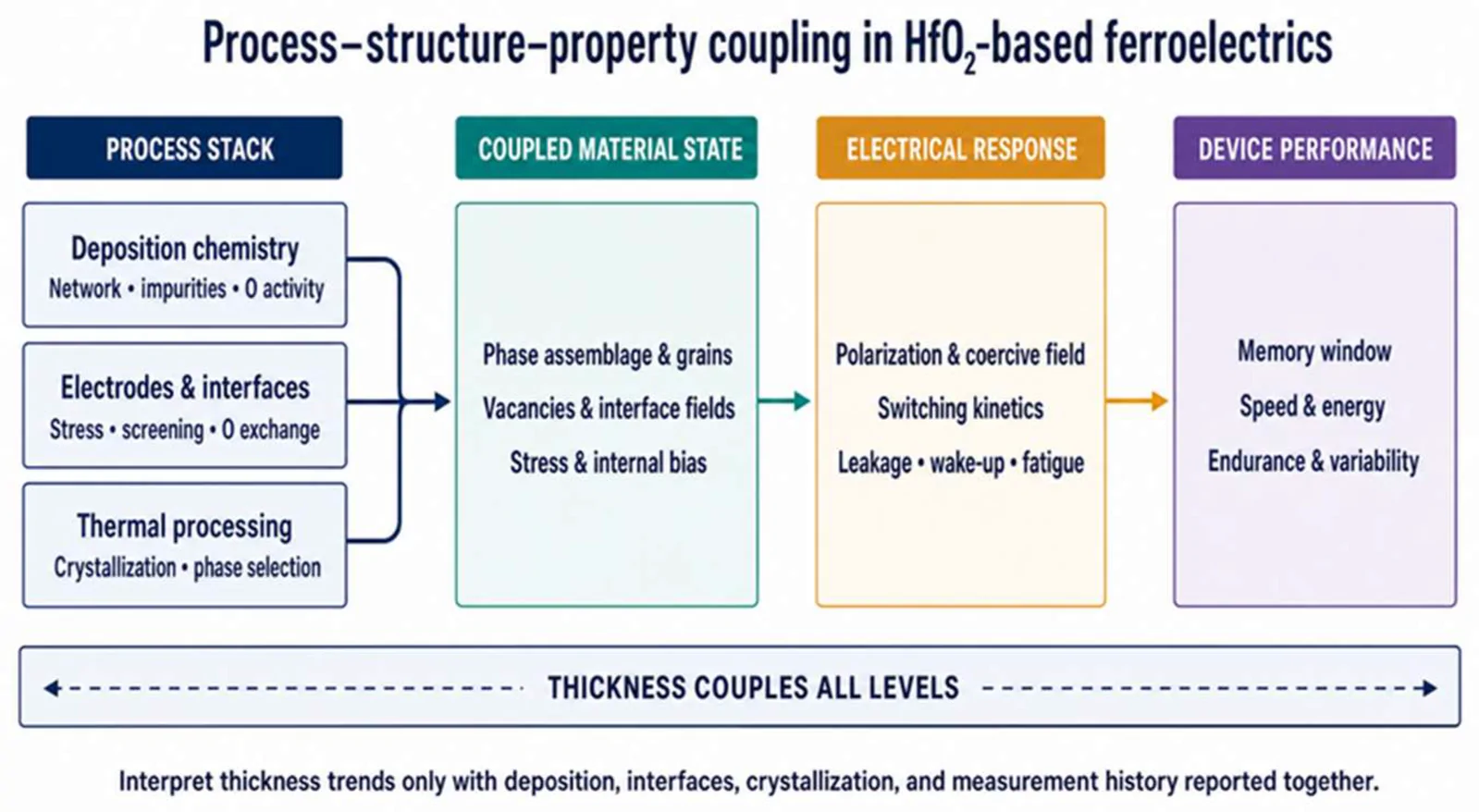
Coupled process–structure–property–device framework for HfO_2_-based ferroelectrics.

**Figure 3:**
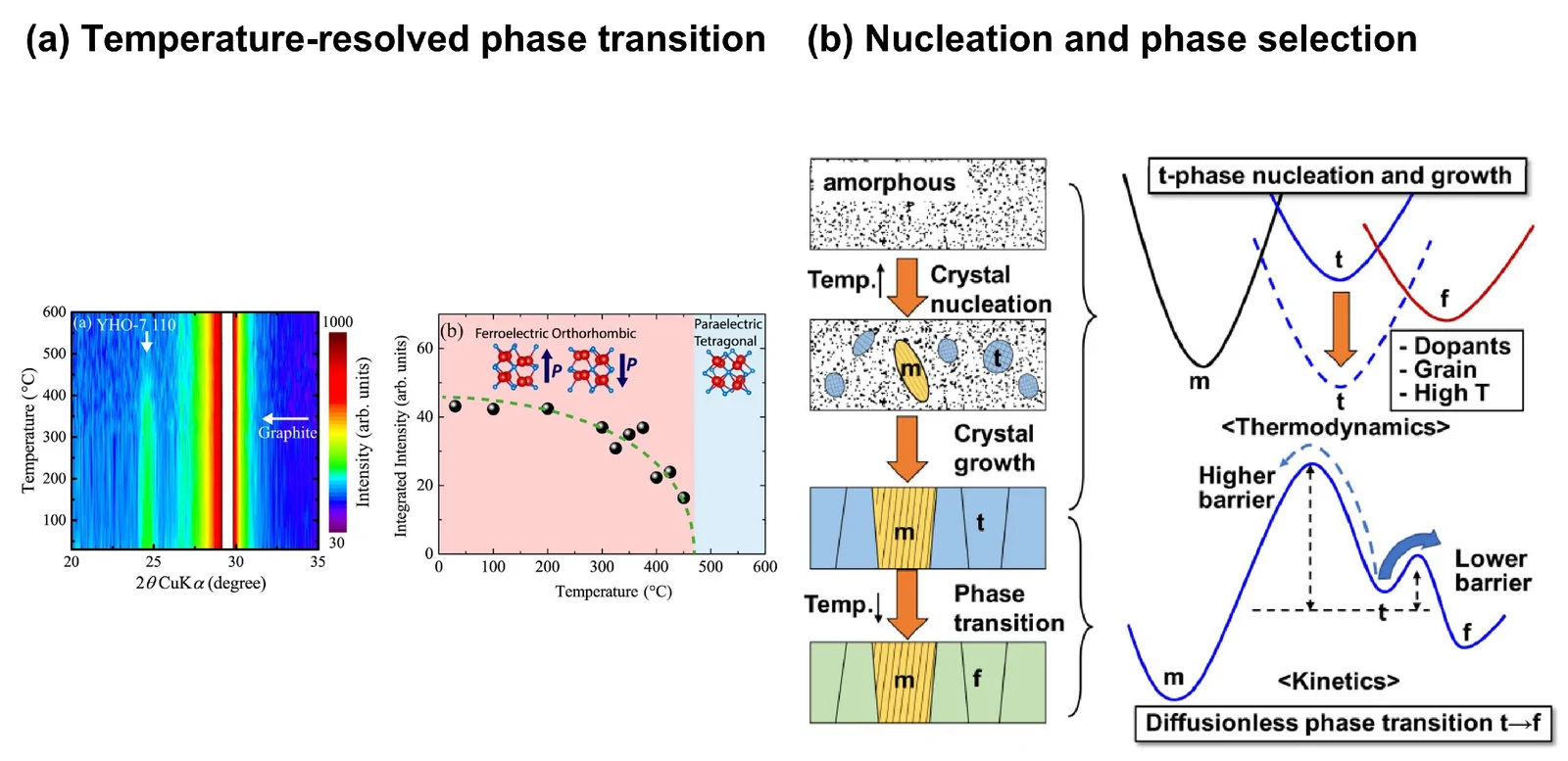
Crystallization pathways and temperature-dependent structural evidence. (**a**) Temperature-resolved diffraction and the integrated intensity of a transition-sensitive reflection in Y-doped hafnia. Reprinted from Ref. [45]. (**b**) Nucleation, growth, and phase-selection mechanisms. Reprinted from Ref. [58]. The original labels within (**a**) identify its diffraction map and reflection-intensity plot, respectively.

**Figure 4:**
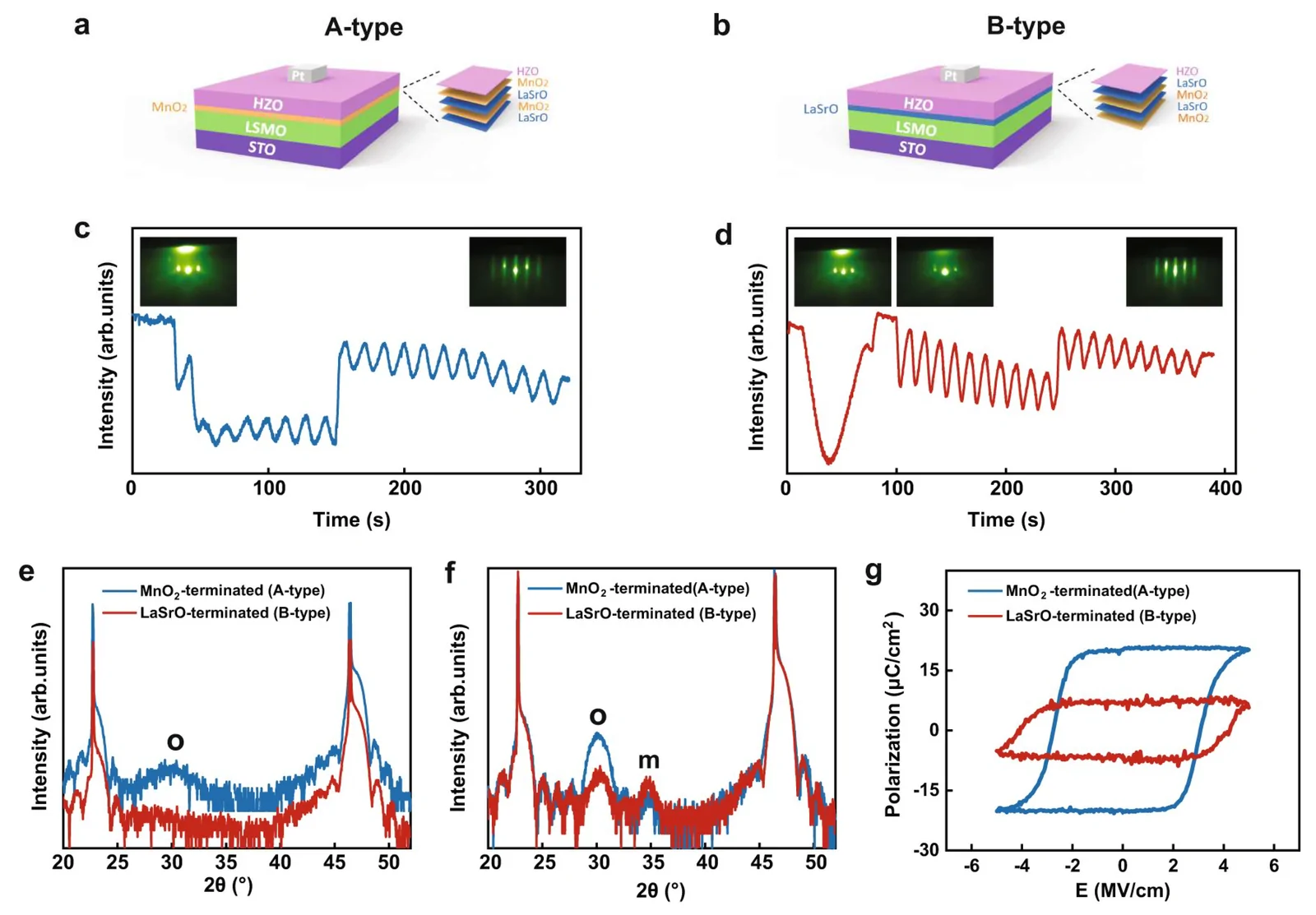
Interface termination as a control of epitaxial HZO. (**a**) A-type heterostructure with MnO_2_ interface termination; (**b**) B-type heterostructure with LaSrO interface termination; (**c**) RHEED intensity oscillations during A-type growth; (**d**) RHEED intensity oscillations during B-type growth; (**e**) XRD patterns of 1.5-nm-thick HZO heterostructures; (**f**) XRD patterns of 8-nm-thick HZO heterostructures, with “o” and “m” indicating the orthorhombic and monoclinic phases, respectively; (**g**) PUND polarization–electric field loops of 8-nm-thick A-type and B-type HZO heterostructures. Reprinted from Ref. [71].

**Figure 5:**
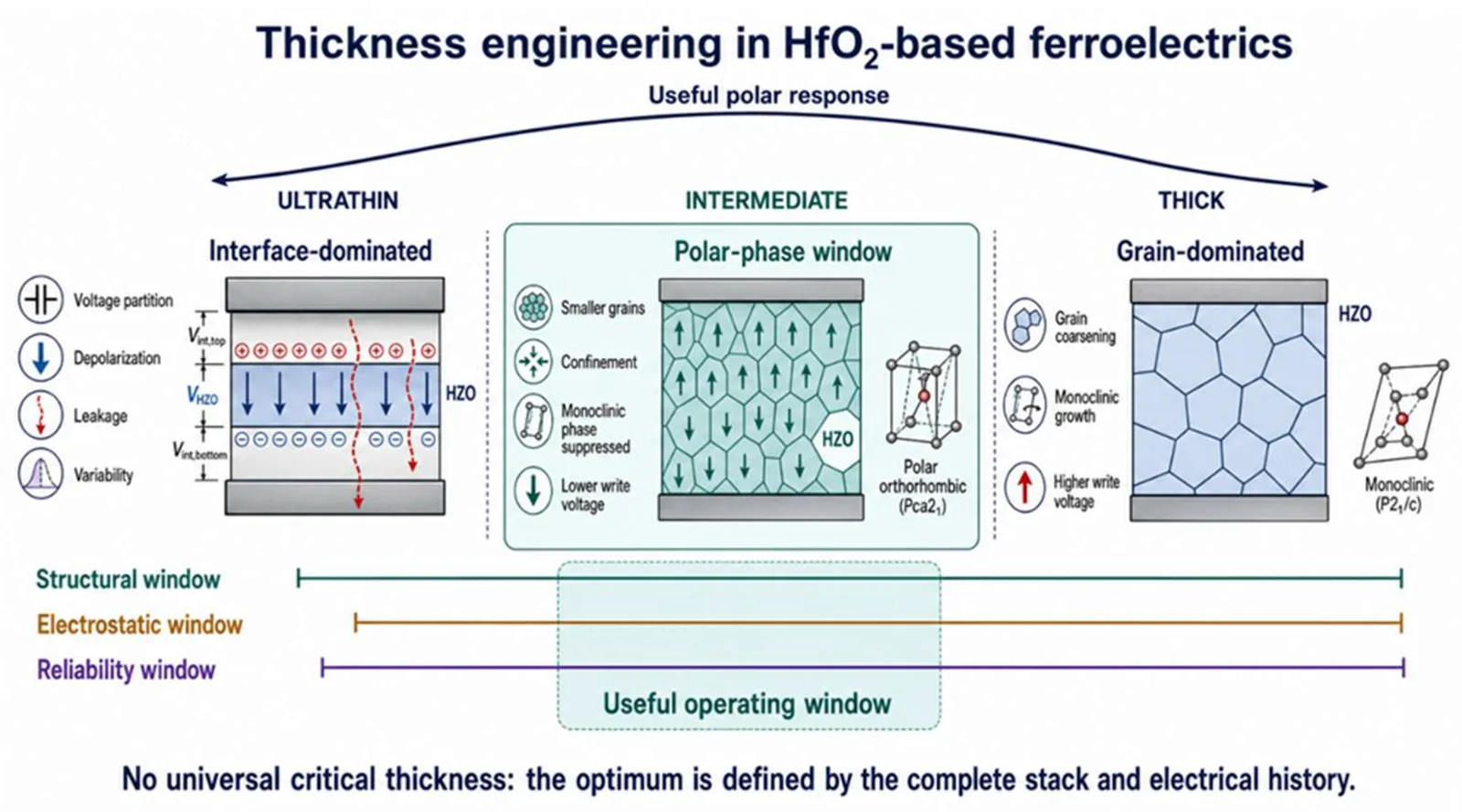
Thickness-dependent operating window in HfO_2_-based ferroelectrics.

**Figure 7:**
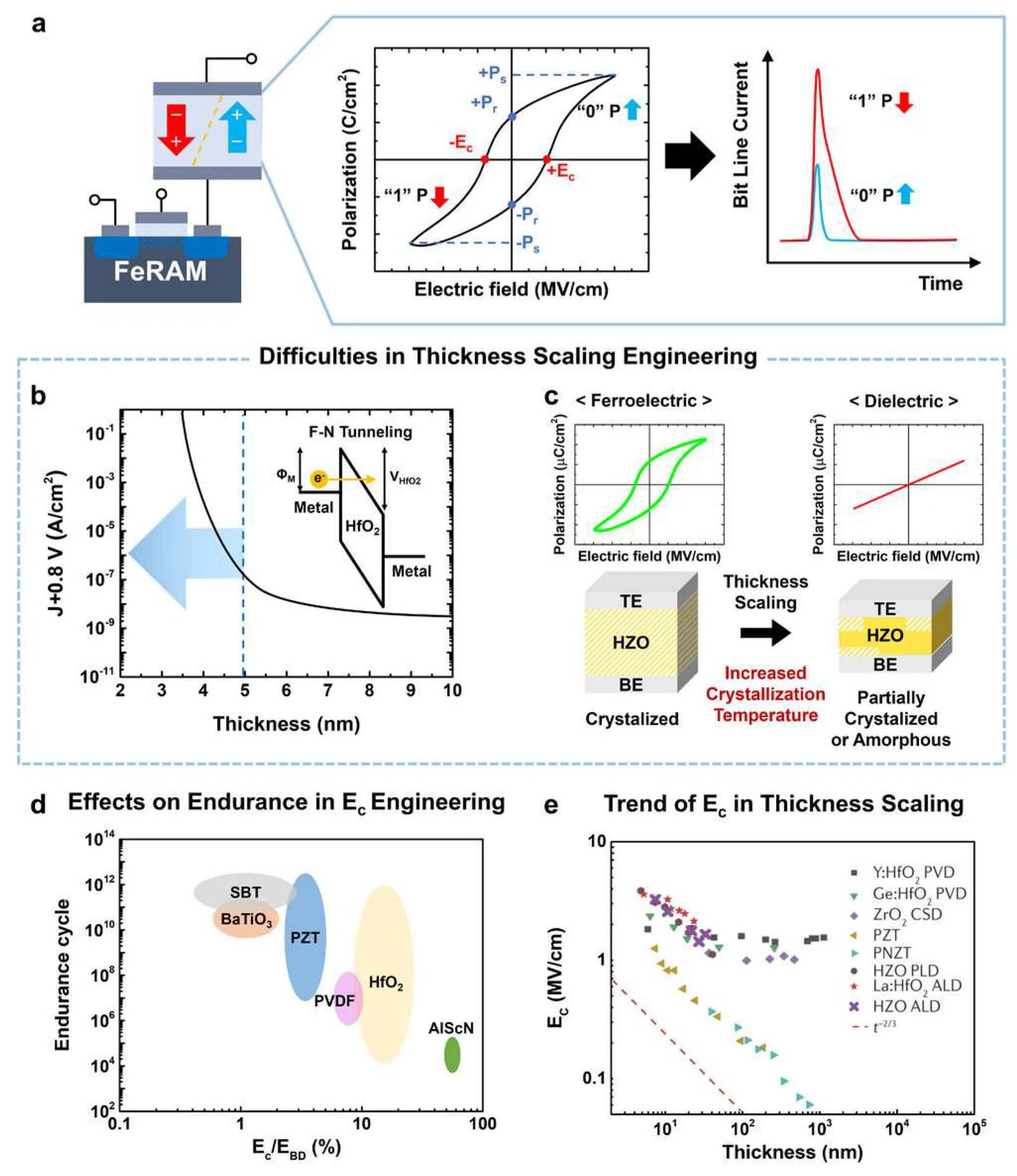
Thickness-scaling constraints: (**a**) FeRAM polarization states and readout; (**b**) leakage current density versus film thickness, with a tunneling schematic; (**c**) crystallization constraints upon thinning; (**d**) reported endurance versus the coercive-to-breakdown-field ratio; and (**e**) coercive field versus film thickness for representative ferroelectrics. Reprinted from Ref. [88].

**Figure 8:**
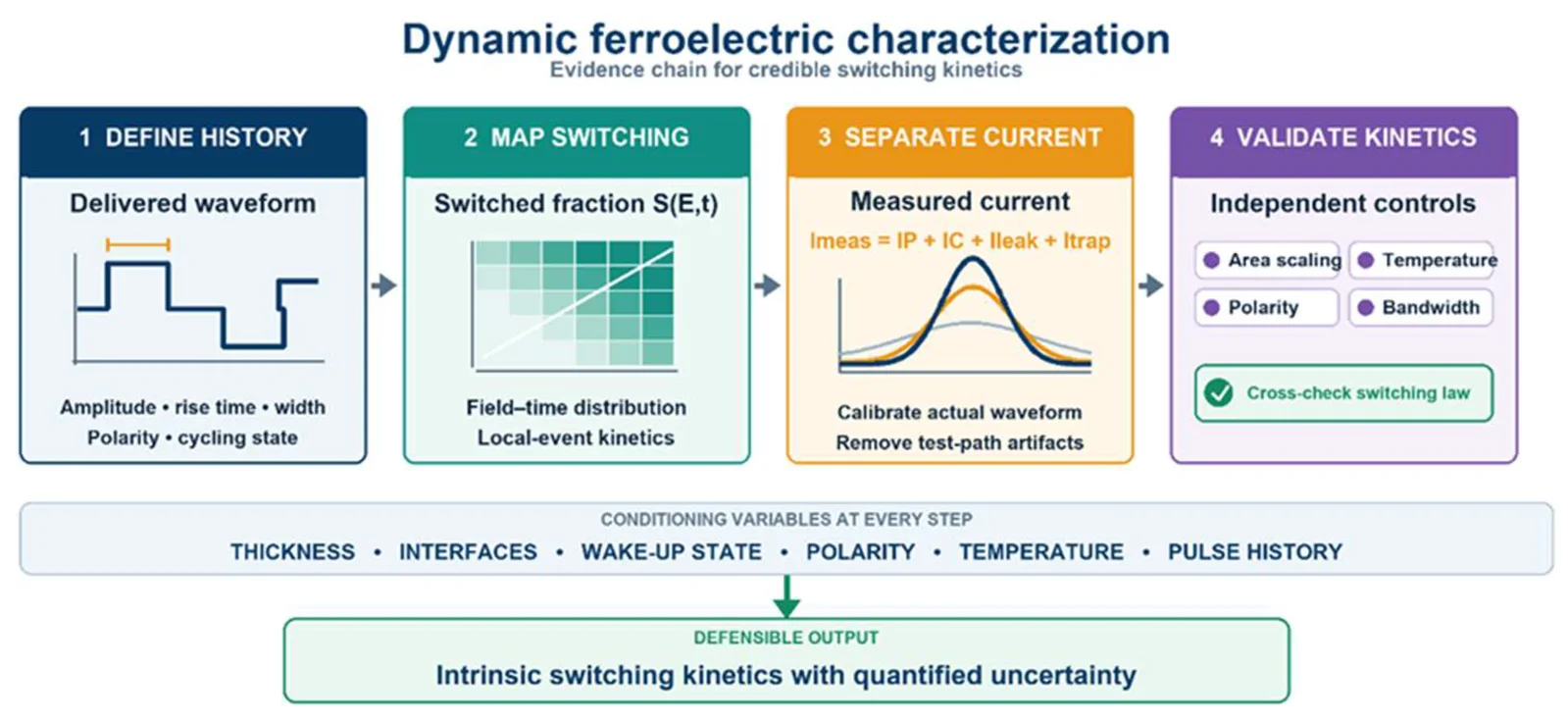
Evidence chain for dynamic ferroelectric characterization.

**Figure 9:**
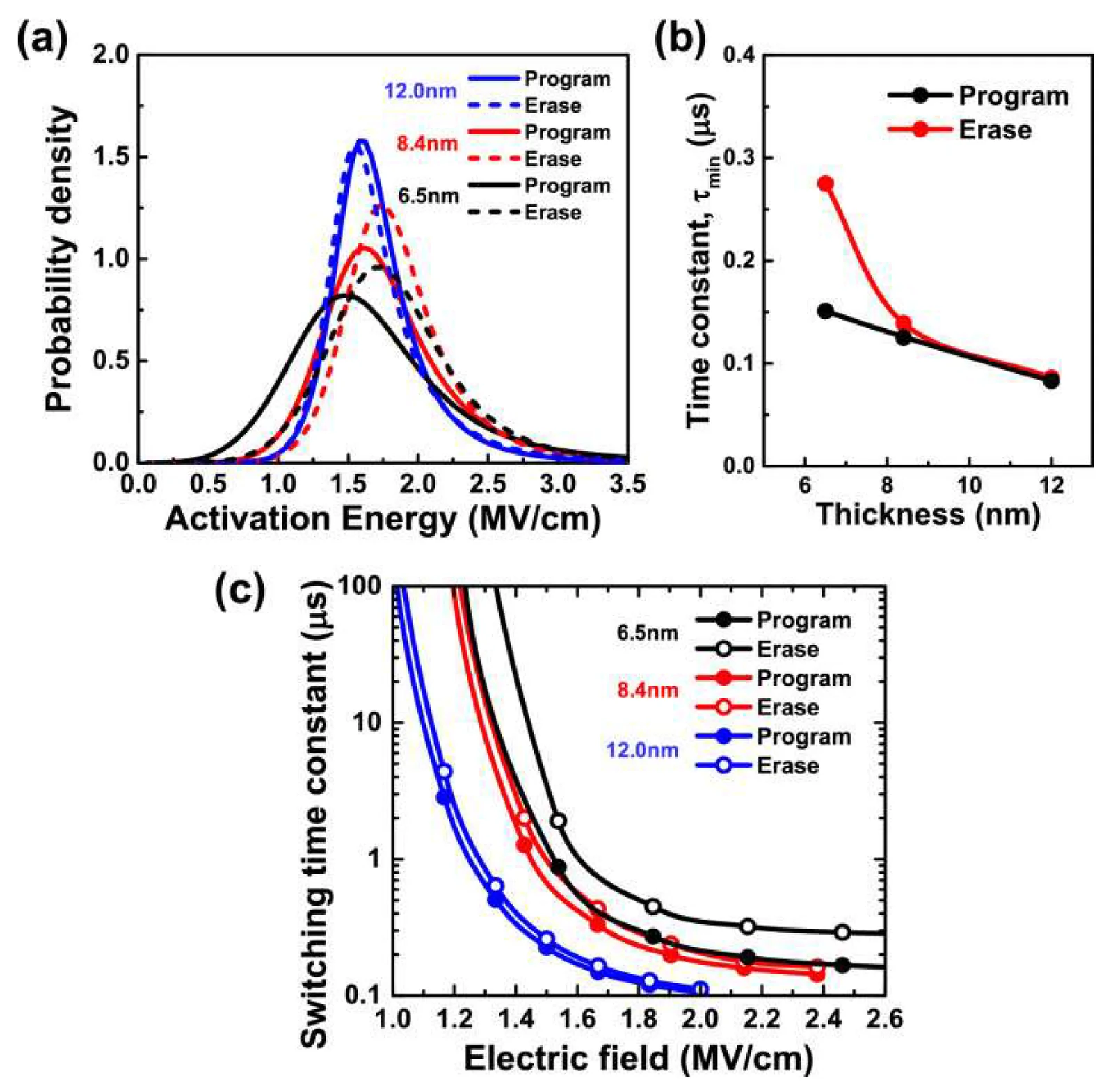
Thickness-dependent switching kinetics in HZO: (**a**) extracted effective activation-field distributions; (**b**) fitted minimum switching times; and (**c**) calculated average switching time constants versus ferroelectric field. Program and erase results are distinguished for each thickness. Reprinted from Ref. [58].

**Figure 10:**
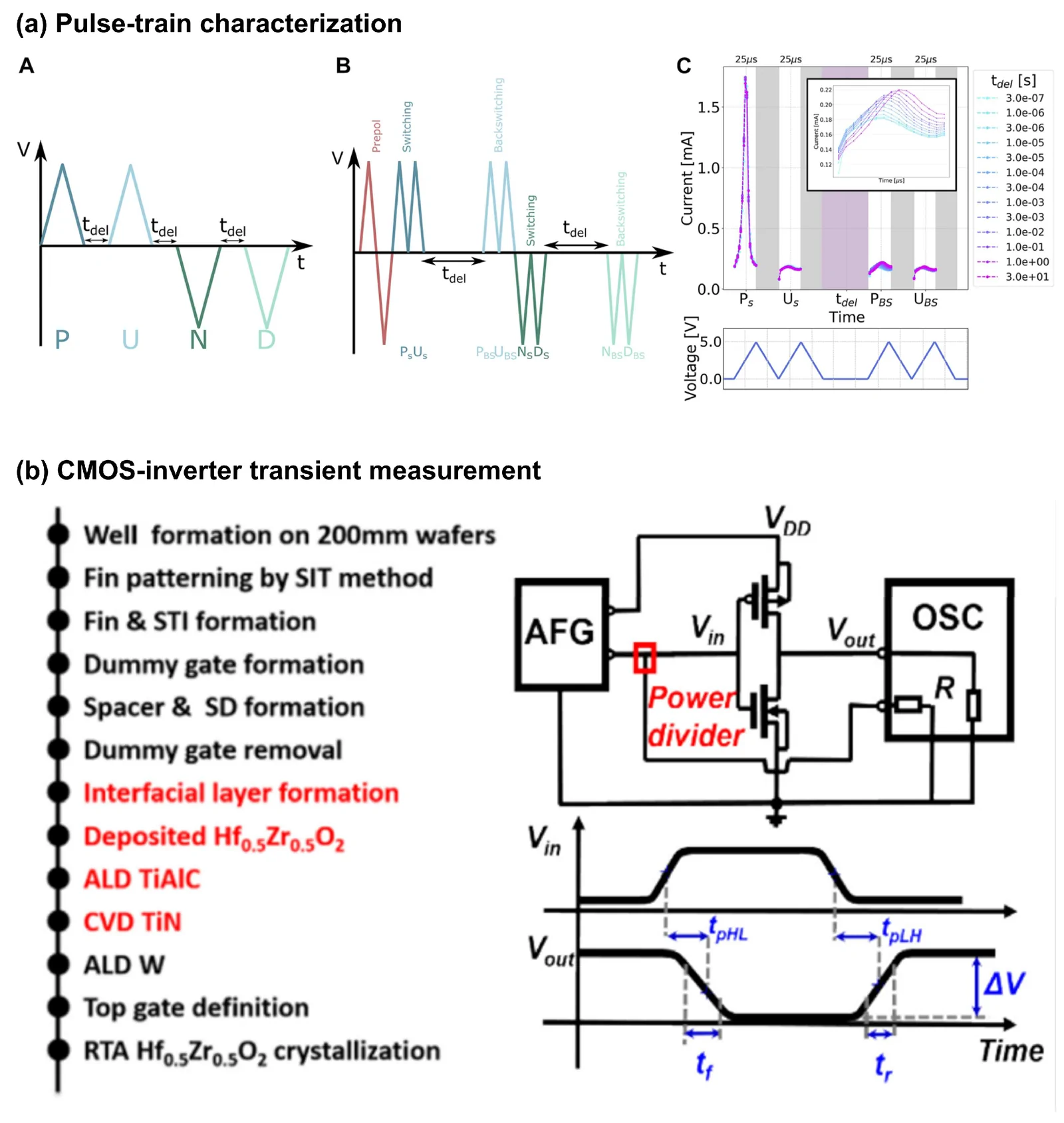
Pulse-train characterization and CMOS-inverter transient measurement. (**a**) A: conventional PUND; B: modified sequence measuring switching and back-switching after a zero-bias delay; C: corresponding delay-dependent current traces. Blue/green identify positive/negative polarity; in B, darker/lighter pairs measure switching/back-switching, and red denotes prepolarization. In C, cyan-to-magenta traces encode increasing delay time as indicated by the legend; purple shading marks the zero-bias delay. Reprinted from Ref. [23]. (**b**) CMOS-inverter fabrication flow and transient measurement scheme. Reproduced with permission from Ref. [98].

**Figure 11:**
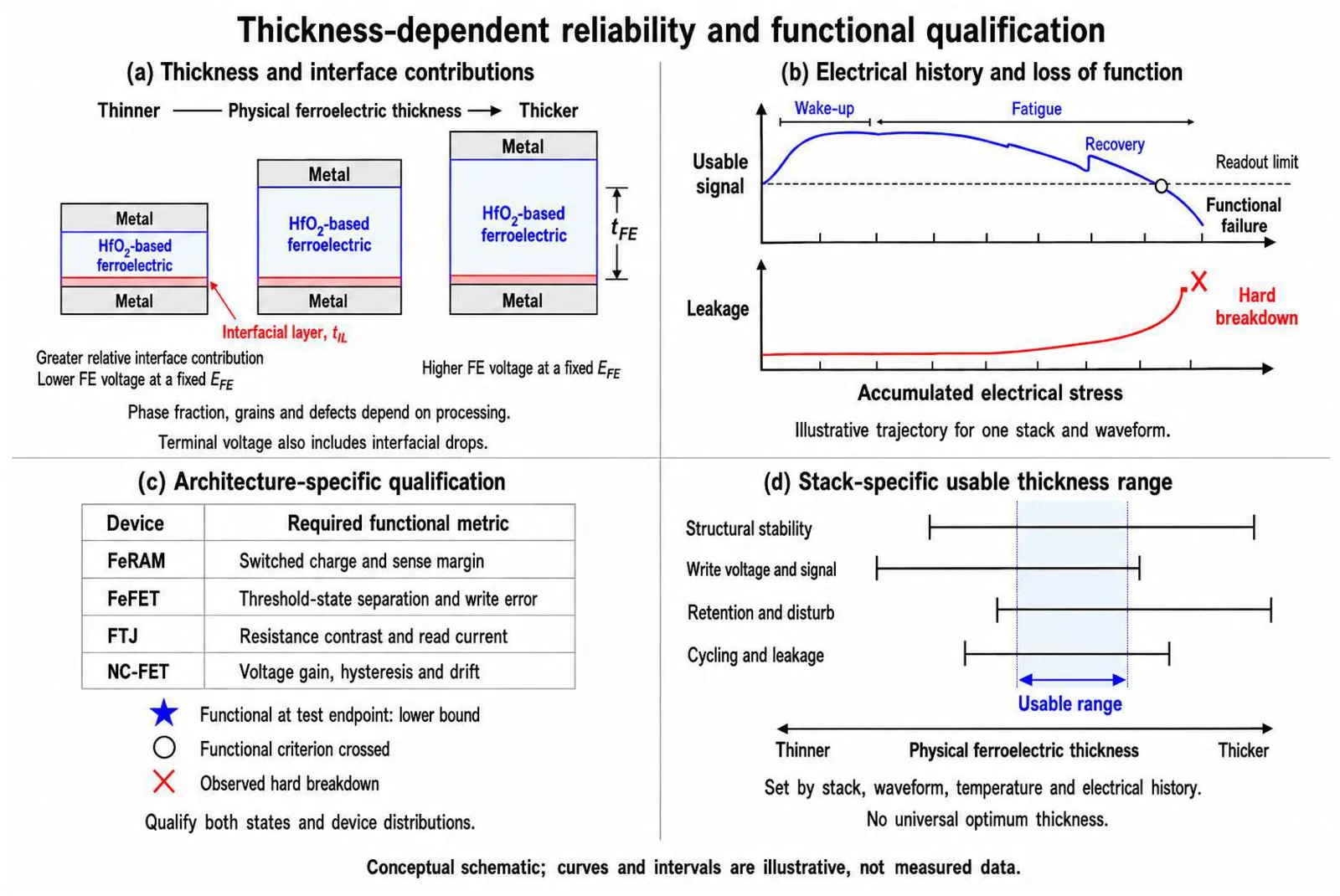
Thickness-dependent reliability and functional qualification of HfO_2_-based ferroelectrics. (**a**) Thickness and interface contributions. (**b**) Evolution of usable signal and leakage under electrical stress. (**c**) Device-specific qualification criteria; stars denote lower bounds on functional lifetime when the stated acceptance criterion is still satisfied at the test endpoint; open circles denote functional failure, and crosses denote hard breakdown. (**d**) Joint constraints defining the stack-specific usable thickness range.

**Figure 12:**
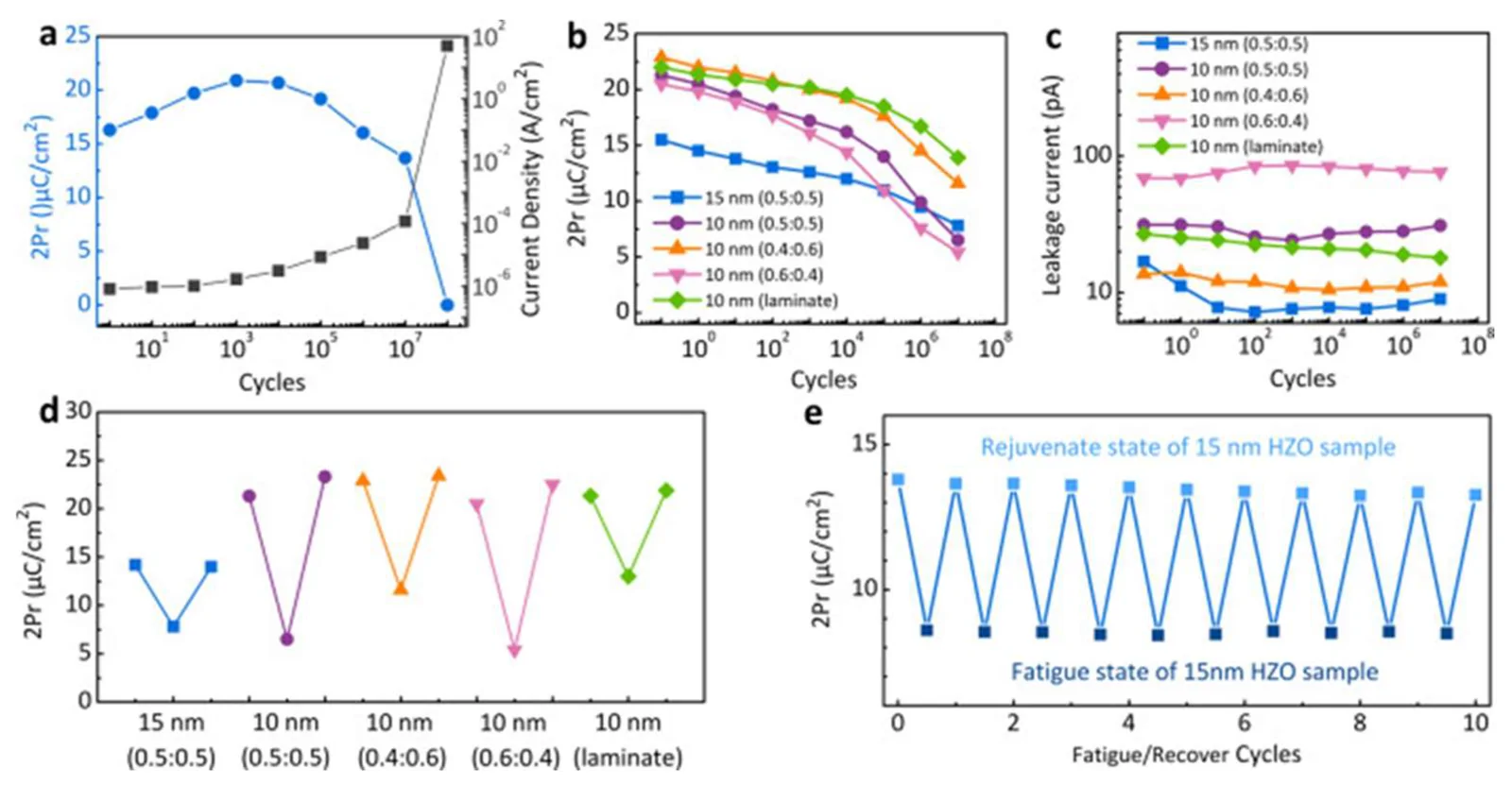
Cycling induced degradation and recovery: (**a**) 2Pr and leakage evolution through wake-up, fatigue, and breakdown; (**b**) 2Pr and (**c**) leakage under low-field cycling for different film compositions and thicknesses; (**d**) polarization before fatigue, after fatigue, and after higher-field recovery; and (**e**) repeated fatigue/recovery cycles. Reprinted from Ref. [104].

**Figure 13:**
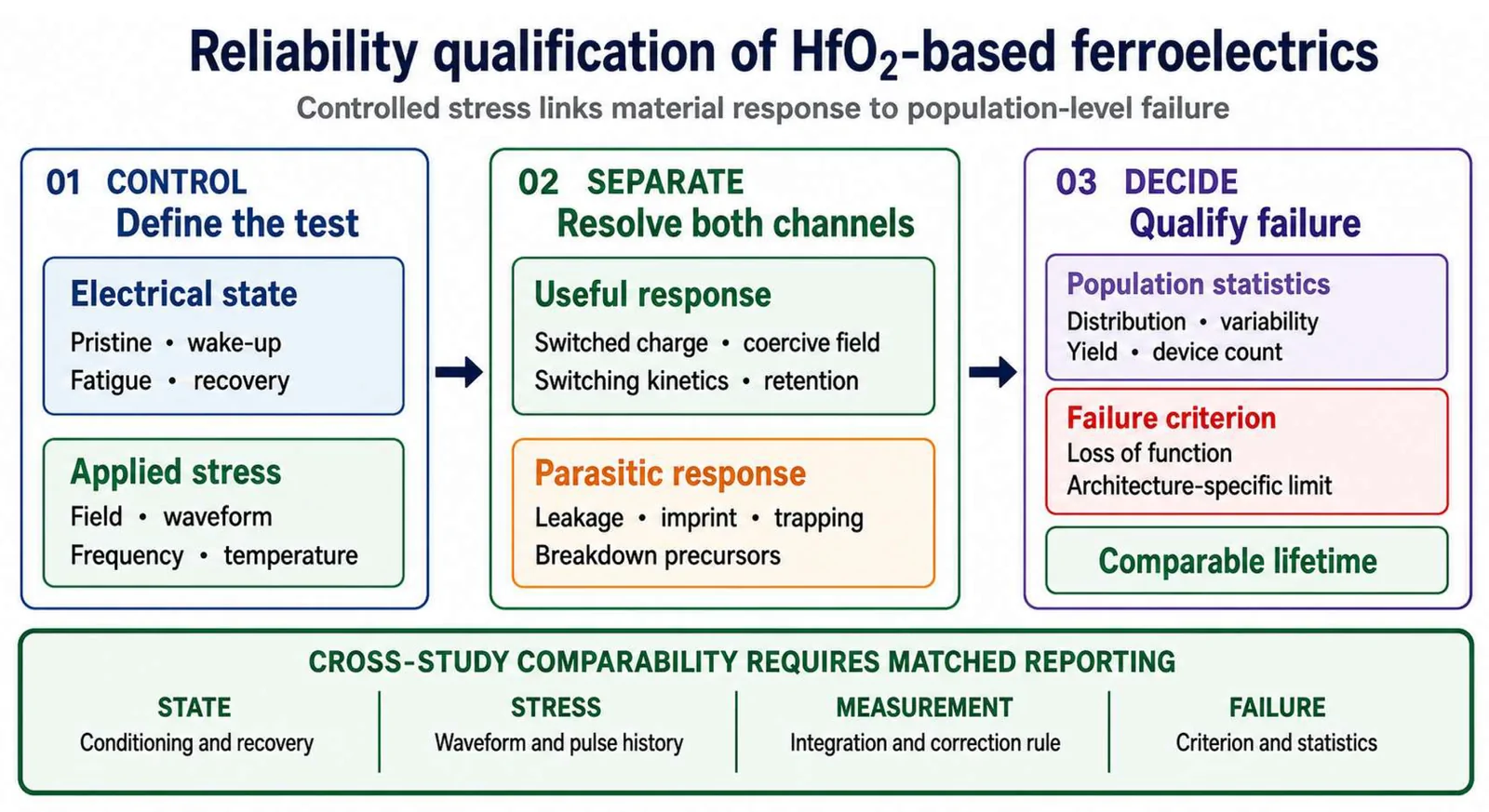
Reliability qualification from electrical state, stress history, useful signal, parasitic response, and device statistics.

**Figure 14:**
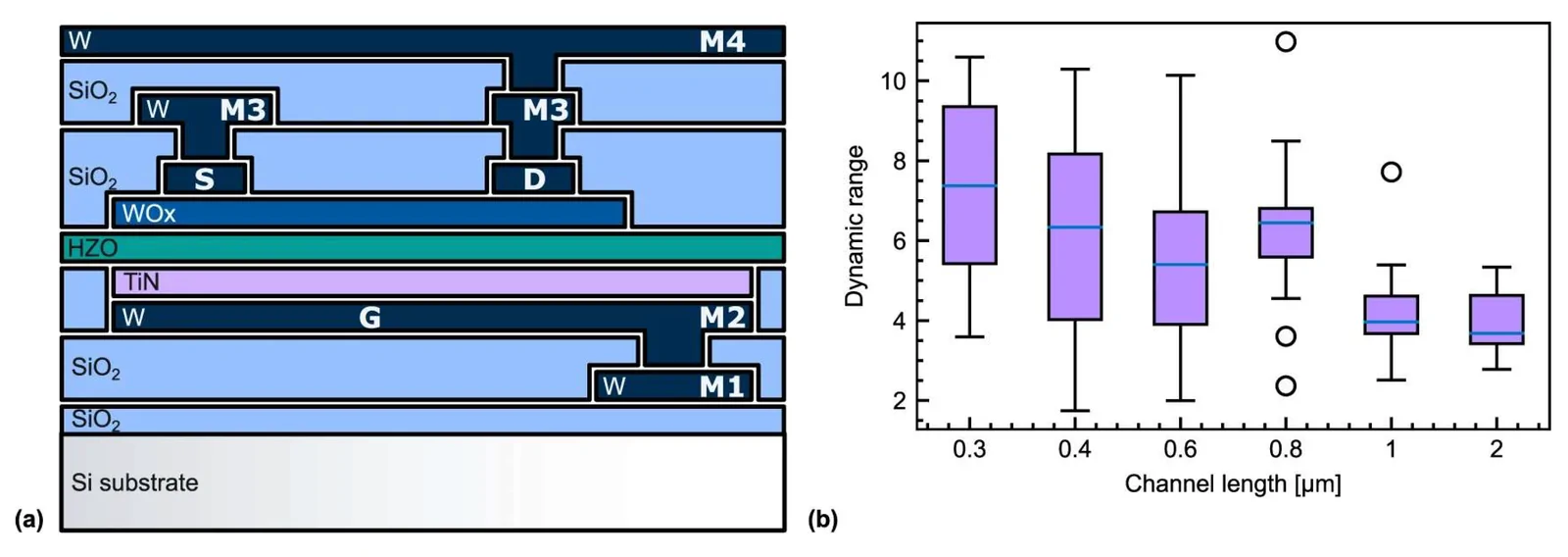
HZO-based synaptic FeFET: (**a**) device structure with a WO_x_ channel; and (**b**) conductance dynamic-range distributions versus channel length. Channel length, rather than ferroelectric thickness, is varied. Reprinted from Ref. [117].

**Table 1:** Deposition controls and their thickness-dependent consequences.

Route	Controls to Retain	Effect of Thickness	Advantage and Limitation
ALD [37,42,43]	Precursor/oxidant; pulse/purge; cycle sequence; temperature	Initial nucleation, continuity, intermixing, and cap-dependent crystallization	Conformal thickness control; nominal cycles do not establish active thickness
Reactive sputtering [44]	Target; power; pressure; oxygen fraction; substrate bias	Bombardment damage, density, and stress evolve with growth time	High throughput; plasma and oxygen conditions complicate process transfer
PLD [45,48,49,50]	Target; fluence; oxygen pressure; temperature; epitaxial template	Strain relaxation, orientation, and interface contributions change	Useful for controlled orientation; thermal budget and conformality limit transfer
CSD [46,47]	Solution; coating; pyrolysis; atmosphere; repeated anneals	Continuity, shrinkage, and cumulative crystallization history	Flexible composition; ultrathin continuity and repeated heating require control

**Table 2:** Representative thickness-dependent evidence for HfO_2_-based ferroelectrics and limitations on cross-study comparison. Stack drawings are schematic. E1 and E2 denote the Pt/CeO_2−x_/HZO/LSMO and Pt/LSMO/CeO_2−x_/HZO/LSMO stacks, respectively. These labels distinguish the two interface designs reported in Ref. [26].

Study	Device Stack/Physical FE Thickness	State, Protocol and Thickness Relevant Result	Constraint on Cross-Study Comparison
Sawabe et al. [25]	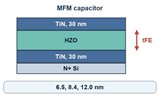	After conditioning, low-frequency Pr is similar, but thinner HZO switches more slowly and has a broader activation-field distribution (100 ns–10 ms).	Measured thickness series: 6.5/8.4/12 nm. Device size and bandwidth were checked down to 100 ns
Zhou et al. [26]	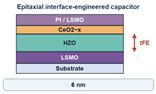	6 nm HZO and ~0.8 nm CeO_2−x_; 25 μm diameter. E1: cycling beyond 2 × 10^10^. E2 with added top LSMO: <5% polarization loss at 10^11^ and switching retained at 10^12^ cycles.	HZO growth at 600 °C; bottom LSMO at 700 °C. E1 and E2 are distinct stacks. Retention beyond 10 years is projected from 358 K tests, not directly observed.
Park et al. [28,51]	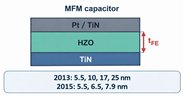	Ref. [45]: Deposition at 280 °C; Pr ≈ 16 μC cm^−2^ for 10 nm at 600 °C. The orthorhombic fraction decreases as thickness increases. Ref. [75]:1 kHz P-E; 2Pr rises from 7.5/17.2/24.0 to 26.9/35.7/36.8 μC cm^−2^.	Ref. [45]: N_2_ RTA for 30 s at 400–800 °C. Thickness and annealing jointly determine phase assemblage; Ref. [75]: N_2_ RTA for 10 s: 600 °C for 5.5 nm, 500 °C for thicker films. Annealing co-varies with thickness.
Cheema et al. [31]	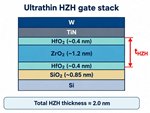	Structural, C-V and 500 ns Q-V data support a mixed-ferroic gate stack with ~0.65 nm EOT.	Designed HfO_2_/ZrO_2_ sequencing on SiO_2_/Si; not equivalent to random alloy HZO in an MFM capacitor.
Cheema et al. [30]	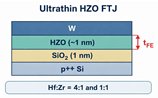	SHG, PFM, SCM, spectroscopy/diffraction and FTJ tests support switchable polar distortion at ~1 nm.	The study includes Hf:Zr ratios of 4:1 and 1:1. The FTJ measurements use a W/HZO/SiO_2_/Si stack; PFM-derived field estimates and local retention observations refer to different measurement configurations.
Lyu et al. [81]	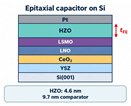	PLD at 800 °C; 20 μm electrode diameter. Bipolar square cycling at 100 kHz: 2.5 V reaches 10^11^ cycles without breakdown (final 2Pr ≈ 6.6 μC cm^−2^); breakdown near 10^10^ at 3.0 V and 10^9^ at 3.5 V.	At 2.5 V, pristine 2Pr ≈ 27 μC cm^−2^ and projected 10-year 2Pr > 12 μC cm^−2^. Voltage changes the endpoint at fixed thickness. This cycling lower bound does not imply degradation-free operation; extrapolated retention is not direct observation.
Hsain et al. [85]	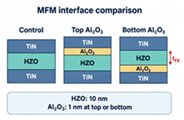	Sequential ALD; 800 °C, 30 s N_2_ anneal; At 3.5 MV cm^−1^ and 100 kHz, control breakdown ~2 × 10^4^; both interlayer variants reach a cycling lower bound of 10^8^ cycles without observed breakdown. Top interlayer 2Pr ≈ 42 μC cm^−2^.	Interface comparison at fixed HZO thickness. Both variants fatigue; peak polarization and last cycling point are different states. The reported field is not a directly measured internal HZO field.

**Table 3:** Common electrical metadata for the same studies.

Study	Electrical Area	Switching/Coercive Field	Leakage	Retention
Sawabe et al. [25]	Circular capacitor: radius ≈15 μm.	Pulse-dependent switching maps measured over 100 ns–10 ms. The operational coercive field corresponds to 50% of the normalized switched polarization and depends on pulse width and polarity.	Leakage suppression is discussed qualitatively; a numerical leakage current density is NR.	NR
Zhou et al. [26]	Circular electrode: diameter 25 μm.	CeO_2−x_-capped HZO: reported Ec ≈ 2.9 MV cm^−1^; do not transfer this value to the added-LSMO stack.	J–E curves for the different stacks; no common single leakage value is used here.	Pt/LSMO/CeO_2−x_/HZO/LSMO: retention beyond 10 years is projected from measurements at 358 K; this is an extrapolation, not a direct ten-year observation.
Park et al. [28,51]	Refs. [28,51]: circular electrode, diameter 300 μm.	Ref. [51]: P–E curves. Ref. [28]: P–E loops measured at 1 kHz and post-wake-up (E_c_) plotted against inverse thickness; the frequency dependence is discussed through a model, rather than presented as a measured frequency sweep dataset.	Ref. [51]: I–V characteristics are reported in the Supplementary Material; no single leakage-current-density value is extracted here. Ref. [28]: a numerical leakage current-density value is NR.	NR
Cheema et al. [31]	Area of the selected MOS/MIM capacitors: NR.	Pulsed Q–V characterization uses 500 ns pulses. A single coercive field value applicable to the selected 2 nm mixed-ferroic HfO_2_–ZrO_2_–HfO_2_ gate stack is NR.	Gate leakage curves/benchmark at Vg − Vfb = −1 V.	Two-state nonvolatile retention: NR.
Cheema et al. [30]	Area of the selected ~1 nm electrical devices: NR.	Effective (E_c ≈ 2) MV cm^−1^ is estimated from PFM switching voltages using a 1 nm HZO/2 nm SiO_2_ voltage divider model that neglects semiconductor accumulation/depletion. It is not a direct FTJ coercive-field measurement.	FTJ I–V curves; separately quantified parasitic leakage: NR.	Written PFM contrast retained ≥24 h; quantitative electrical two-state retention: NR.
Lyu et al. [81]	Circular electrode: diameter 20 μm.	P–E curves; cycling amplitudes of 2.5, 3.0 and 3.5 V are terminal voltages, not internal coercive fields.	J–E curves; retain field and cycling-state dependence.	Projected 10-year 2Pr > 12 μC cm^−2^ after 2.5 V poling; extrapolated, not directly observed.
Hsain et al. [85]	Circular electrode: diameter 200 μm.	After 10^4^ cycles, reported 2Ec: control 3.3, bottom interlayer 3.8, top interlayer 3.2 MV cm^−1^; these are 2Ec, not Ec.	At +2 V: interlayer variants ~10^−5^ A cm^−2^ after 10^7^ cycles at 3.5 MV cm^−1^; full J–E curves also reported.	NR.

NR = not reported for the specified quantity in the cited study; it does not mean zero or failed performance. “Curves” identifies graphical reporting without digitization into a single comparable value. Geometry is retained as reported; for circular electrodes, area = πd^2^/4. Applied cycling voltage, nominal field, Ec, and 2Ec are not interchangeable. Retention entries distinguish direct observation, local PFM contrast, and electrical extrapolation.

**Table 4:** Architecture-specific consequences of changing physical ferroelectric thickness and measurable functional criteria. Trends are conditional on composition, interfaces, processing, and electrical state; no universal thickness or acceptance threshold is implied.

Architecture	Thinner Ferroelectric	Thicker Ferroelectric	Output and Failure Definition
FeCAP/FeRAM	Potentially lower write voltage; interfacial loss and leakage may reduce short-pulse charge	Potentially better insulation; higher voltage; charge grows only if ΔPsw improves	Qsw(twrite) and sense margin; fail below specified minimum at access time
FeFET memory	Better physical scaling; larger fractional interlayer drop, trapping, and depolarization	Possible larger window; increased EOT and program voltage	Retained threshold distributions; fail at margin loss or specified state-overlap probability
FTJ	Higher tunnel current; roughness, parasitic transport, and disturb become critical	Lower current; potentially improved continuity and retention	Read current and TER = (ROFF − RON)/RON at fixed bias; fail outside sensing limits
NC-FET	Changes capacitance matching and EOT; interface sensitivity increases	Stronger nonlinear capacitance contribution; possible voltage and hysteresis penalty	Measured gain, hysteresis, BTI; fail at predefined logic/reliability limits
FeNAND	Reduced radial stack and possible write voltage; disturb and local nonuniformity risk	Higher program/pass voltage; difficult string uniformity	Cell and string state distributions; fail at disturb or multilevel read-margin limits
Associative/logic memory	Possible energy reduction; match-current dispersion and state drift	Possible state margin; higher voltage/energy	Match/mismatch separation after retention and disturb at target array size
Analog/neuromorphic	Possible lower update energy; interface sensitivity and stochastic updates	Possible retention benefit; larger pulse voltage and update cost	Linearity, symmetry, noise, dynamic range; fail at specified state or task error

## Data Availability

No new data were created or analyzed in this study. Data sharing is not applicable.
